# Supplementary material for: Control of seed formation allows two distinct self-sorting patterns of supramolecular nanofibers
Source: Nat Commun. 2020 Aug 14;11:4100. doi: 10.1038/s41467-020-17984-x (PMC7428048; doi:10.1038/s41467-020-17984-x)
Supplement: Supplementary file 1 — Supplementary Information [file 41467_2020_17984_MOESM1_ESM.pdf]

Supplementary information

Control of seed formation allows two distinct self-sorting patterns of supramolecular nanofibers

Kubota et al.

Supplementary Methods

General. Unless stated otherwise, all commercial reagents were used as received. Thin layer chromatography (TLC) was performed on silica gel $60F_{254}$ (Merck). ^{1}H, ^{13}C, and ^{19}F NMR spectra were obtained on a Varian Mercury 400 and a JEOL JNM-ECZ500, and JMM-ECA600 spectrometer with tetramethylsilane (0 ppm) for ^{1}H NMR, with a residual solvent peak (39.52 ppm) for ^{13}C NMR as the internal references, and sodium trifluoromethanesulfonate (–78.8 ppm) for ^{19}F NMR as the external references. ESI mass spectra were recorded using an Exactive (Thermo Scientific). Reversed-phase HPLC (RP-HPLC) was carried out on a Hitachi Chromaster system equipped with a diode array and YMC-Pack Triart C18 or ODS-A columns. All runs used linear gradients of acetonitrile (ACN) containing 0.1% trifluoroacetic acid (TFA) and 0.1 % aqueous TFA. 4-(1,3-dioxolan-2-yl)benzoic acid, **Phos-MecycC$_5$**, **NP-Alexa647** and **NBD-cycC$_6$** were synthesized as previously reported.[1–3] The images of confocal laser scanning microscopy (CLSM) were acquired by a LSM 800 equipped with an Airyscan unit (Carl Zeiss Microscopy). Plan-Apochromat objectives (63×, 1.40 numerical aperture, oil immersion, Zeiss) was used. For all of the time-lapse CLSM imaging, Definite Focus 2 (Carl Zeiss) was employed to compensate for the sample drift. 3D CLSM movies were made by Imaris 9.5 (Bitplane). Pearson's correlation coefficients were calculated by Coloc 2 program in Fiji.[4] Rheological measurements were carried out using MCR-502 (Anton Paar).

Preparation of Ald-F(F)F and BnOx-F(F)F hydrogels. A suspension of an **Ald-F(F)F** or **BnOx-F(F)F** powder in 100 mM MES, pH 6.0 was heated by a heating gun (PJ-206A1, Ishizaki) until dissolving. The resultant hot solution was cooled to room temperature (rt) and incubated for 24 h. The state (gel or sol) of the sample was judged by the tube inversion test. The assay conditions were referred in the figure captions.

Hydrogelation through in situ formation of BnOx-F(F)F. A suspension of an **Ald-F(F)F** powder (4.3 mM) in 100 mM MES, pH 6.0 was heated by a heating gun until dissolving. The resultant hot solution was cooled to rt and incubated for 1 h. To this resultant solution, a solution of *O*-benzylhydroxylamine (43 mM in 100 mM MES, pH 6.0) was added and incubated for 1 h. The state (gel or sol) of the sample was judged by the tube inversion test. The assay conditions were referred in the figure

captions.

In situ formation of BnOx-F(F)F from Ald-F(F)F gel. A suspension of an **Ald-F(F)F** powder (17.3 mM) in 100 mM MES, pH 6.0 was heated by a heating gun until dissolving. The resultant hot solution was cooled to rt and incubated for 1 h. To this resultant hydrogel, a 10× solution of *O*-benzylhydroxylamine (173 mM in 100 mM MES, pH 6.0) was added and incubated for 1 h. The hydrogel was analyzed by rheological and HPLC analyses.

CLSM imaging in the oxime-formation protocol. The suspension of **Ald-F(F)F** (4.3 mM) and **NP-Alexa647** (4.0 μM) with/without **Phos-MecycC$_5$** (2.4 mM) and **NBD-cycC$_6$** (4.0 μM) in 100 mM MES, pH 6.0 was heated by a heating gun until dissolving. After cooling to rt, the resultant mixture (18 μL) was transferred to a glass bottom dish (Matsunami) and incubate at rt for 1 h in the presence of water to avoid dryness. To the resultant solution, a solution of *O*-benzylhydroxylamine (43.2 mM, 1 μL in 100 mM MES, pH 6.0) or buffer was added. After incubation for 1 h, CLSM imaging was conducted.

Preparation of the suspension of BnOx-F(F)F and Phos-MecycC$_5$. The suspension of **BnOx-F(F)F** (4.3 mM) and **Phos-MecycC$_5$** (2.4 mM) with/without **NP-Alexa647** (4.0 μM) and **NBD-cycC$_6$** (4.0 μM) in 100 mM MES, pH 6.0 was heated by a heating gun until dissolving. The resultant mixture was cooled to rt and incubated at rt for 24 h. The state (gel or sol) of the sample was judged by the tube inversion test. The obtained suspension (containing fluorescent probes) was moved to a glass bottom dish and observed by CLSM imaging.

Mixing hot solutions of BnOx-F(F)F and Phos-MecycC$_5$. The suspensions of **BnOx-F(F)F/NP-Alexa647** (8.6 mM and 8.0 μM) and **Phos-MecycC$_5$/NBD-cycC$_6$** (4.8 mM and 8.0 μM) were separately heated by a heating gun until dissolving. The equal volume of the resultant hot solutions were immediately mixed and incubated at rt for 1 h. The state (gel or sol) of the sample was judged by the tube inversion test. The obtained suspension (containing fluorescent probes) was moved to a glass bottom dish and observed by CLSM imaging.

CLSM imaging in the oxime-exchange protocol. The suspension of **Ald-F(F)F** (17.3 mM) and **NP-Alexa647** (4.0 μM) with **Phos-MecycC$_5$** (2.4 mM) and **NBD-cycC$_6$** (4.0 μM) in 100 mM MES, pH 6.0 was heated by a heating gun until dissolving. After cooling to rt, the resultant mixture (10 μL) was transferred to a glass bottom dish (Matsunami) before gelation. After incubation at rt for 1 h in the presence of water to avoid dryness, CLSM imaging was conducted. To the resultant hydrogel, a solution of carboxymethoxylamine (208 mM, 1 μL in 100 mM MES, pH 6.0) or buffer was added. After incubation for 4 h, CLSM imaging was conducted. Subsequently, *O*-benzylhydroxylamine (300 mM, 2.3 μL in 100 mM MES, pH 6.0) or buffer was added to the resulting solution. After incubation at rt for 48 h, CLSM imaging was conducted.

Suspension of CaOx-F(F)F and Phos-MecycC$_5$. The suspension of **CaOx-F(F)F** (17.3 mM) and **Phos-MecycC$_5$** (2.4 mM) with/without **NP-Alexa647** (4.0 μM) and **NBD-cycC$_6$** (4.0 μM) in 100 mM MES, pH 6.0 was heated by a heating gun until dissolving. The resultant mixture was cooled to rt and incubated at rt for 1 h. The state (gel or sol) of the sample was judged by the tube inversion test. The obtained suspension (containing fluorescent probes) was moved to a glass bottom dish and observed by CLSM imaging.

Trial for conversion from BnOx-F(F)F to CaOx-F(F)F. To a solution of **Ald-F(F)F** (4.3 mM) with/without **NP-Alexa647** (4.0 μM) were added a 10× solution of O-benzylhydroxylamine (43 mM). The resultant mixture was incubated at 30 ºC for 1 h. A 10× solution of carboxymethoxylamine (430 mM) was added to the hydrogel and incubated at 30 ºC for 24 h. The resulting samples were analyzed by HPLC analysis and CLSM imaging. The state (gel or sol) of the sample was judged by the tube inversion test.

Slow cooling down of BnOx-F(F)F and Phos-MecycC$_5$ mixture. The suspension of **BnOx-F(F)F** (4.3 mM) and **Phos-MecycC$_5$** (2.4 mM) with/without **NP-Alexa647** (4.0 μM) and **NBD-cycC$_6$** (4.0 μM) were heated by a heating gun until dissolving. The resultant hot solutions were incubated on a heating plate at 100 ºC for 5 min, then the

setting temperature was changed from 100 ºC to 30 ºC, and the sample was incubated for 30 min on the heating plate. The state (gel or sol) of the sample was judged by the tube inversion test. The sample was analyzed by CLSM imaging.

HPLC analysis of the hydrogels. The preparation of the hydrogels and solutions was described above. The samples (133 μL) were dissolved by cold DMF (800 μL) and a DMSO solution of fluorescein (internal standard, 3 mM, 90 μL). The resultant mixture was filtered with membrane filter (diameter: 0.45 μm), and then analyzed by RP-HPLC (column: YMC-Triart C18, A:B = 30:70 to 90:10 for 30 min, A: CH_3CN containing 0.1% TFA, B: H_2O containing 0.1% TFA).

Rheological analysis. The preparation of the hydrogel was the same as described above. The resultant disk-shaped hydrogels (*ca.* 10 mm) were carefully took out from the PDMS mold and put onto the stage of a rheometer (MCR-502, Anton Paar) with a parallel plate geometry. Strain sweep data were obtained using shear mode at a frequency of 10 rad/s, and linear dynamic viscoelasticity were measured in shear mode at 0.3 or 1.0% strain amplitude for frequency sweep.

Determination of nanofiber elongation velocity. The elongation distance of the peptide-type nanofiber was estimated by comparing the successive two images of the time-lapse imaging with Fiji. The velocity was calculated by dividing the elongation distance by interval of time-lapse imaging. 50 and 120 elongated nanofibers were randomly selected (supplementary Fig. 6 and 40, respectively). The histograms were depicted by using Kaleidagraph 4.5 (Synergy Software).

Organic syntheses

Ald-F(F)F

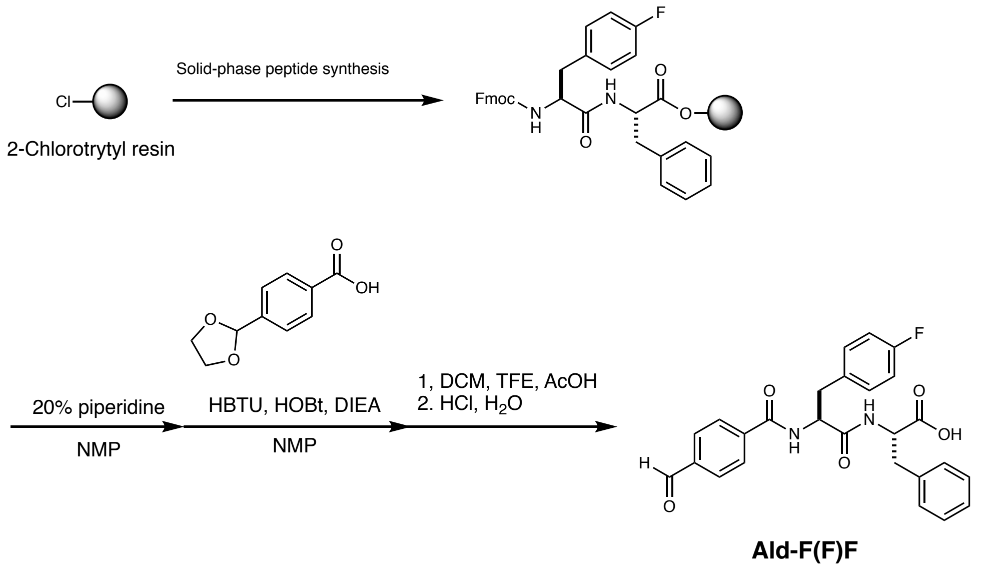

Ald-F(F)F was prepared by Fmoc solid-phase peptide synthesis using a commercially available 2-chlorotrityl resin (105 mg, 150 μmol, 1.0 eq). The condensation reaction was carried out in the presence of Fmoc-protected amino acids (3.0 eq), 2-(1*H*-benzotriazol-1-yl)-1,1,3,3-tetramethyluronium hexafluorophosphate (HBTU: 3.0 eq), 1-hydroxybenzotriazole hydrate (HOBt·H_2O: 3.0 eq) and diisopropylethylamine (DIEA: 6.0 eq) in 1-methyl-2-pyrrolidinone (NMP). Removal of Fmoc protecting group was performed using NMP solution containing 20% piperidine. After removal of the Fmoc group on the terminal amino group, the resulting free amino group was allowed to react with 4-(1,3-dioxolan-2-yl)benzoic acid[1] (3.0 eq) in the presence of HBTU (3.0 eq), HOBt•H_2O (3.0 eq), and DIEA (6.0 eq) in NMP. Finally, cleavage of the compound from the resin were performed using a cocktail (7:2:1 CH_2Cl_2/trifluoroethanol (TFE)/AcOH) at rt for 1.5 h. Deprotection of the crude peptide was carried out in 1:3 mixture of 1 M HCl aq. and TFA at rt for 3 h. The crude product was purified by RP-HPLC (column: YMC ODS-A, solvent gradient: A:B = 40:60 to 70:30 for 30 min, A: CH_3CN with 0.1% TFA, B: H_2O with 0.1% TFA) to give **Ald-F(F)F** as a white solid (20.0 mg, 43.2 μmol, 28%).

^{1}H NMR (600 MHz, DMSO-d_6, rt): δ 10.07 (s, 1H), 8.77 (d, J = 9.0 Hz, 1H), 8.39 (d, J = 9.0 Hz, 1H), 7.97 (d, J = 8.4 Hz, 2H), 7.93 (d, J = 8.4 Hz, 2H), 7.36 (dd, J = 8.4, 6.0 Hz, 2H), 7.24 (m, 4H), 7.18 (m, 1H), 7.07 (dd, J = 8.4 Hz, 2H), 4.75 (m, 1H), 4.48 (m, 1H), 3.08 (m, 2H), 2.93 (m, 2H).

^{13}C NMR (150 MHz, DMSO-d_6, rt): δ 192.9, 172.7, 171.1, 165.3, 160.6 (J_{CF} = 241.18 Hz), 138.9, 137.8, 137.4, 134.3 (J_{CF} = 3.46 Hz), 131.0 (J_{CF} = 8.29 Hz), 129.3, 129.2, 128.2, 128.1, 126.5, 114.8 (J_{CF} = 21.42 Hz), 54.6, 53.6, 36.6, 36.1.

^{19}F NMR (470 MHz, DMSO-d_6, rt): δ –117.427 (m).

HR-FTMS (ESI): Calcd. For $[M-H]^-$: m/z = 461.1521; found: 461.1507

BnOx-F(F)F

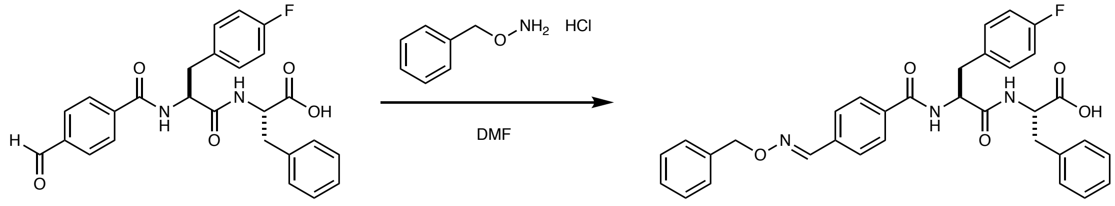

Ald-F(F)F BnOx-F(F)F

A solution of *O*-benzylhydroxylamine hydrochloride (2.6 mg, 16.2 μmol, 1.0 eq) and **Ald-F(F)F** (7.5 mg, 16.2 μmol, 1.0 eq) in DMF (100 μL) was stirred at room temperature for 24 h. The solvent was removed under reduced pressure to give **BnOx-F(F)F** as a white solid (8.8 mg, 15.5 μmol, 96 %).

^{1}H NMR (600 MHz, DMSO-d_6, rt): δ 8.61 (d, J = 10.8 Hz, 1H), 8.36 (d, J = 9.6 Hz, 1H), 8.34 (s, 1H), 7.79 (d, J = 9.6 Hz, 2H), 7.65 (d, J = 10.2 Hz, 2H), 7.43–7.31 (m, 7H), 7.25–7.16 (m, 5H), 7.06 (dd, J = 8.4 Hz, 2H), 5.20 (s, 2H), 4.72 (m, 1H), 4.47 (m, 1H), 3.07 (m, 2H), 2.93 (m, 2H).

^{13}C NMR (150 MHz, DMSO-d_6, rt): δ 172.7, 171.2, 165.5, 160.9 (J_{CF} = 241.6 Hz), 148.6, 137.43, 137.38, 134.9, 134.47, 134.40, 131.0 (J_{CF} = 7.42 Hz), 129.2, 128.37, 128.29, 128.18, 127.90, 127.88, 126.6, 126.5, 114.7 (J_{CF} = 21.19 Hz), 75.7, 54.5, 53.5, 36.6, 36.1.

^{19}F NMR (470 MHz, DMSO-d_6, rt): δ –117.494 (m).

HR-FTMS (ESI): Calcd. For $[M-H]^-$: m/z = 566.2095; found: 566.2086

CaOx-F(F)F

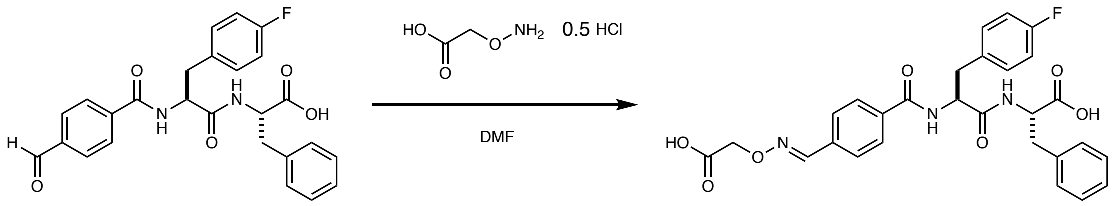

Ald-F(F)F **CaOx-F(F)F**

A solution of carboxymethoxylamine hemihydrochloride (7.09 mg, 64.9 μmol, 1.0 eq) and **Ald-F(F)F** (30.0 mg, 64.9 μmol, 1.0 eq) in DMF (200 μL) was stirred at room temperature for 1 h. The solvent was removed under reduced pressure to give **CaOx-F(F)F** as a white solid (35.5 mg, quant).

^{1}H NMR (600 MHz, DMSO-d_6, rt): δ 8.61 (d, J = 9.0 Hz, 1H), 8.37 (s, 1H), 8.34 (d, J = 7.2 Hz, 1H), 7.80 (d, J = 8.4 Hz, 2H), 7.66 (d, J = 8.4 Hz, 2H), 7.34 (dd, J = 9.0, 6.6 Hz, 2H), 7.24–7.16 (m, 5H), 7.05 (dd, J = 9.0 Hz, 2H), 4.71 (m, 1H), 4.67 (s, 2H), 4.46 (m, 1H), 3.06 (m, 2H), 2.93 (m, 2H).

^{13}C NMR (150 MHz, DMSO-d_6, rt): δ 172.7, 171.2, 170.9, 165.5, 160.8 (J_{CF} = 241.6 Hz), 149.2, 137.4, 135.1, 134.4, 134.1, 131.0 (J_{CF} = 7.63 Hz), 129.2, 128.2, 127.9, 126.8, 126.5, 114.7 (J_{CF} = 20.98 Hz), 70.5, 54.5, 53.5, 36.6, 36.1.

^{19}F NMR (470 MHz, DMSO-d_6, rt): δ –117.488 (m).

HR-FTMS (ESI): Calcd. For $[M-H]^-$: m/z = 534.1682; found: 534.1673

Supplementary Tables

Supplementary Table 1. Abbreviations for reagents used in this research

Abbreviation	Chemical structures
**Ald-F(F)F**	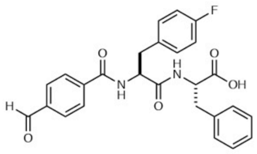
**BnOx-F(F)F**	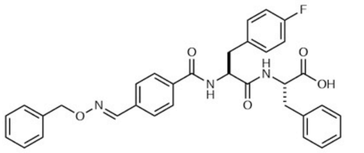
**CaOx-F(F)F**	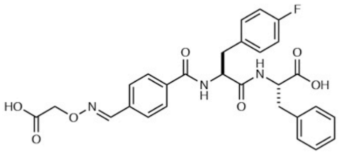
**Phos-MecycC$_5$**	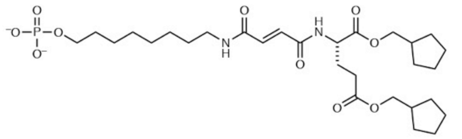
**NP-Alexa647**	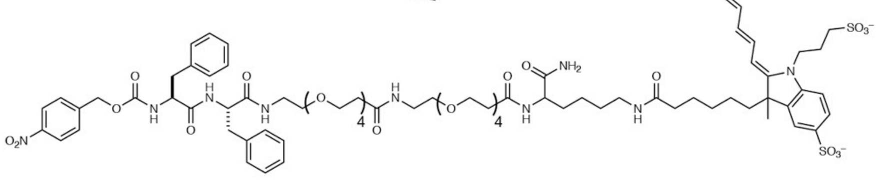
**NBD-cycC$_6$**	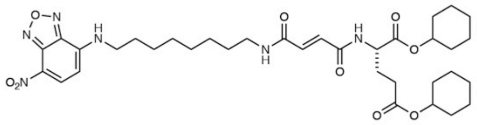

Supplementary Table 2. Critical gelation concentrations of peptide-type hydrogelators

	Ald-F(F)F	BnOx-F(F)F	CaOx-F(F)F
CGC (mM)	8.64	1.32	26.1

Concentrations of the peptide-type hydrogelator
One-step oxime formation protocol: 4.3 mM
Two-step oxime exchange protocol: 17.3 mM

Supplementary Figures

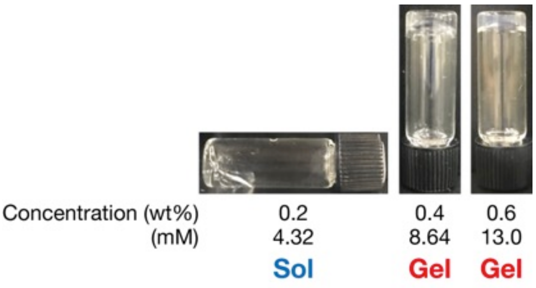

Supplementary Figure 1. Determination of critical gelation concentration of **Ald-F(F)F**. Condition: 100 mM MES, pH 6.0.

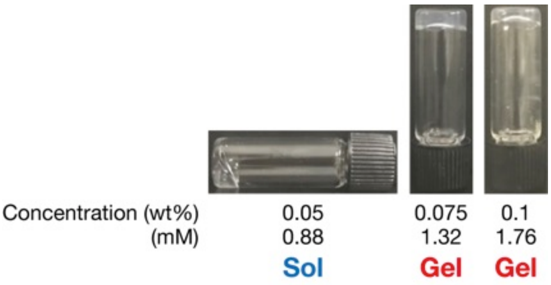

Supplementary Figure 2. Determination of critical gelation concentration of **BnOx-F(F)F**. Condition: 100 mM MES, pH 6.0.

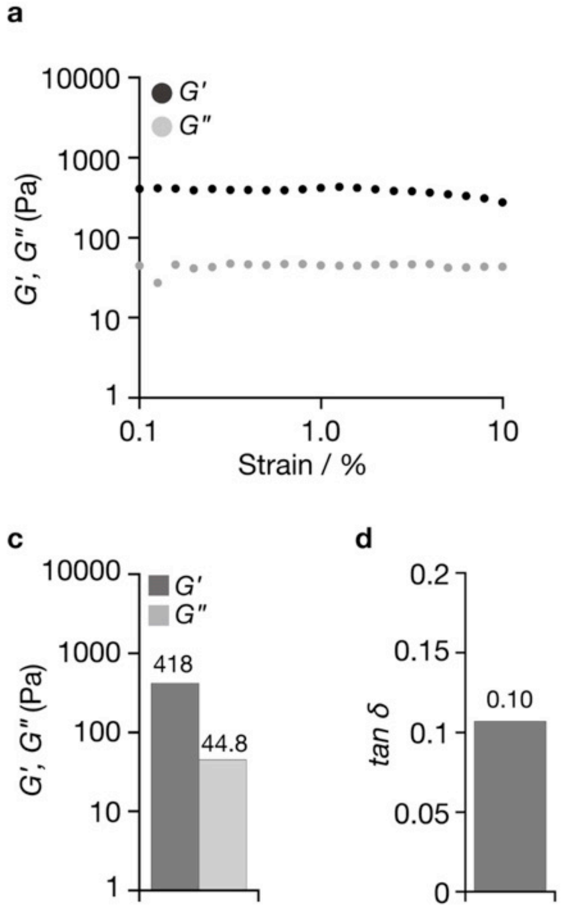

Supplementary Figure 3. (**a**) Strain and (**b**) frequency sweep rheological properties of the hydrogel obtained by addition of *O*-benzylhydroxylamine to **Ald-F(F)F** solution. Frequency for strain sweep: 10 rad/s. Strain amplitude for frequency sweep: 1%. *G*′: storage shear modulus, *G*″: loss shear modulus. (**c**) *G*′, *G*″ and (**d**) tan δ values of the hydrogel. Frequency: 10 rad/s, strain amplitude: 1%. Condition: [**Ald-F(F)F**] = 4.3 mM (0.2 wt%), [*O*-benzylhydroxylamine] = 4.3 mM (1.0 eq) in 100 mM MES, pH 6.0.

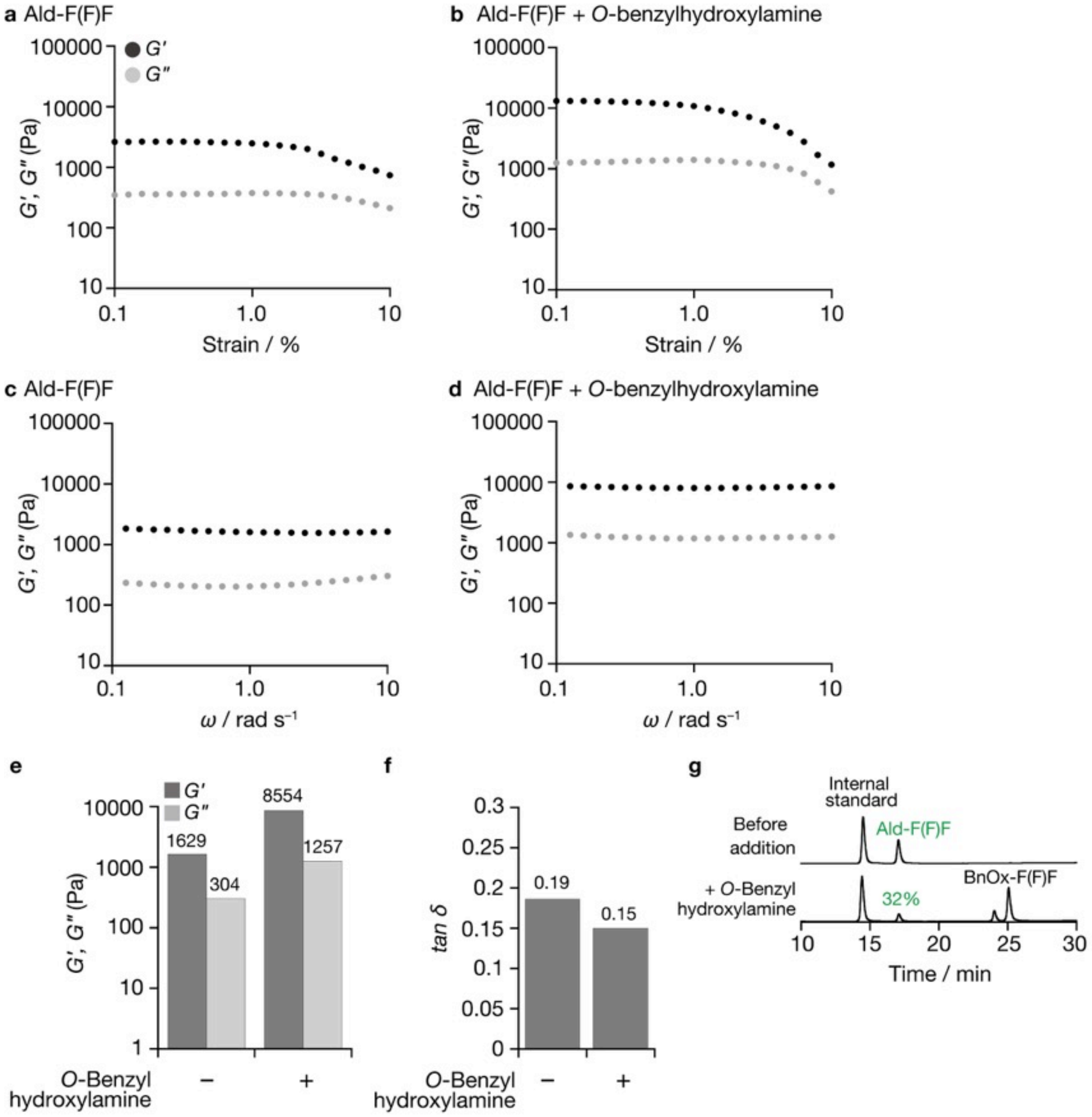

Supplementary Figure 4. (**a**,**b**) Strain and (**c**,**d**) frequency sweep rheological properties of the **Ald-F(F)F** hydrogel (**a**,**c**) before and (**b**,**d**) after addition of *O*-benzylhydroxylamine. Frequency for strain sweep: 10 rad/s. Strain amplitude for frequency sweep: 1%. *G'*: storage shear modulus, *G"*: loss shear modulus. (**e**) *G'*, *G"* and (**f**) tan δ values of the hydrogel. Frequency: 10 rad/s, strain amplitude: 1%. (**g**) HPLC analysis (top) before and (bottom) after treatment of *O*-benzylhydroxylamine. Internal standard: fluorescein. The residual ratio of **Ald-F(F)F** was determined by comparing the integral ratio before *O*-benzylhydroxylamine addition as 100%. Condition: [**Ald-F(F)F**] = 17.3 mM (0.8 wt%), [*O*-benzylhydroxylamine] = 17.3 mM (1.0 eq) in 100 mM MES, pH 6.0.

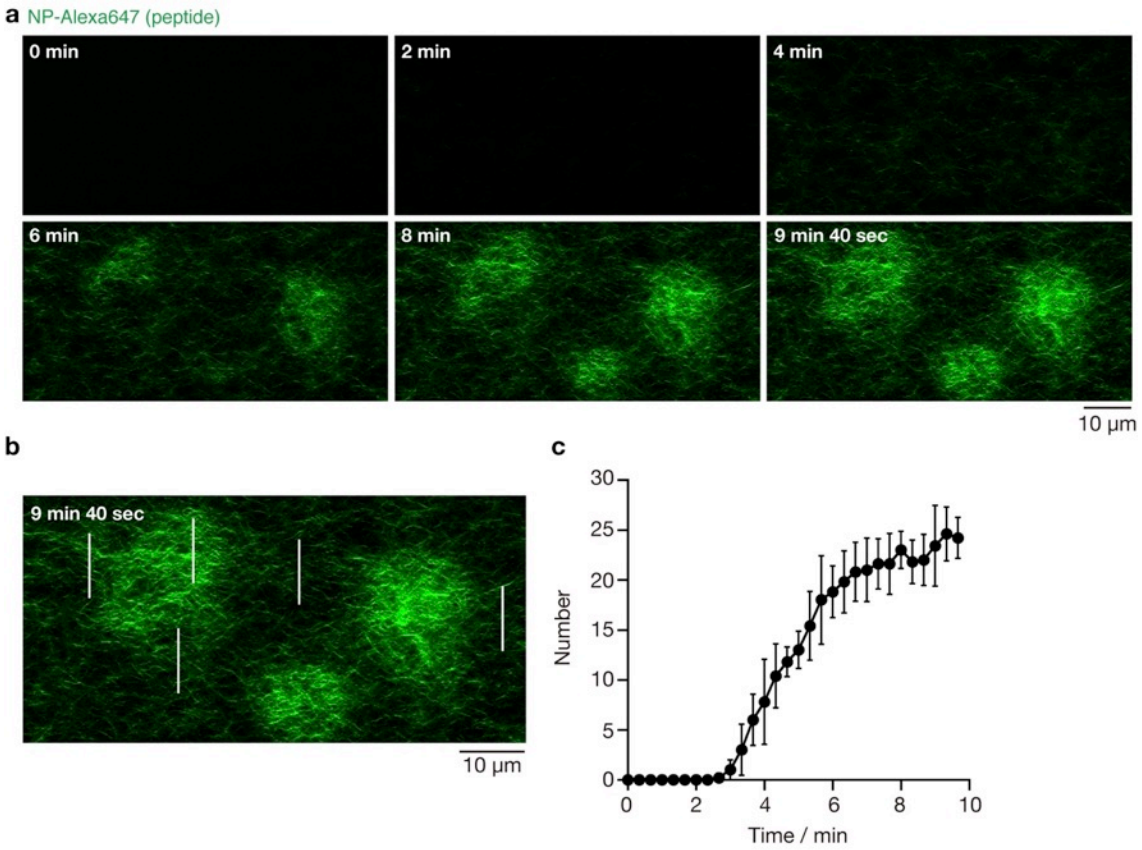

Supplementary Figure 5. (**a**) Time-lapse CLSM images upon addition of *O*-benzylhydroxylamine to **Ald-F(F)F**. (**b**,**c**) Quantitative analysis of the nanofiber formation process. The number of the nanofibers was counted along white lines shown in supplementary Fig. 5b. (**c**) Time course of the average nanofiber number. $n = 5$. The data represent the mean ± standard deviation. Condition: [**Ald-F(F)F**] = 4.3 mM (0.20 wt%), [*O*-benzylhydroxylamine] = 4.3 mM (1.0 eq), [**NP-Alexa647**] = 4.0 μM, 100 mM MES, pH 6.0.

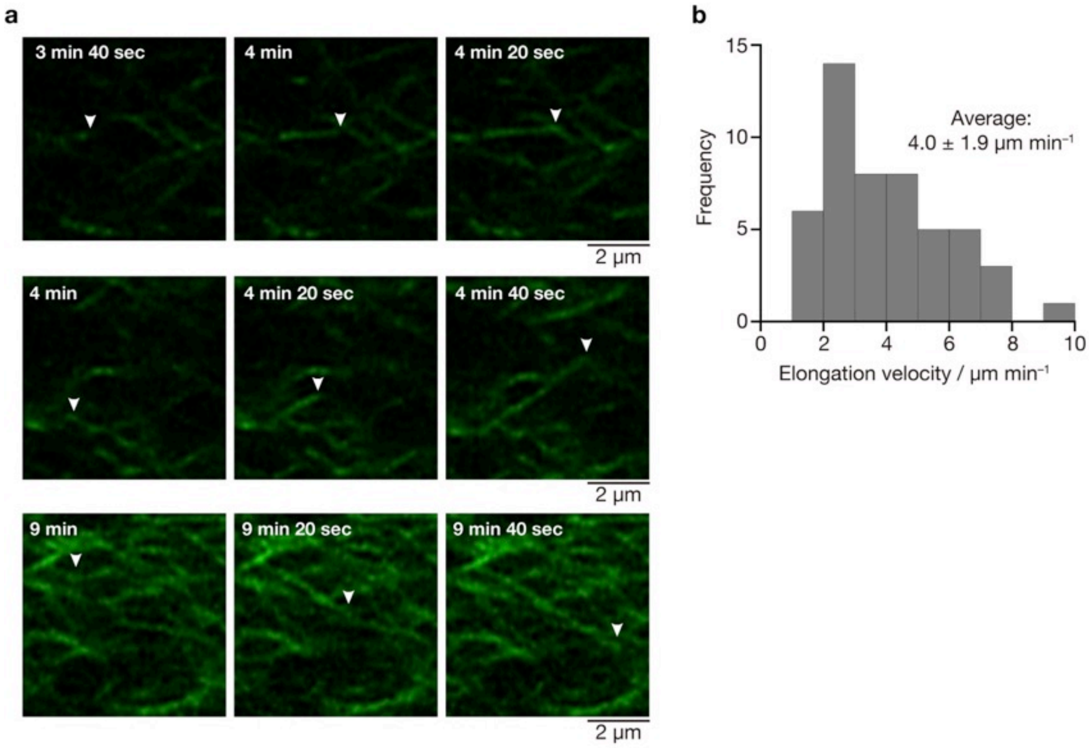

Supplementary Figure 6. (**a**) Formation process of the peptide-type nanofibers. (**b**) Histograms of nanofiber elongation velocity. n = 50. The average value represents the mean ± standard deviation.

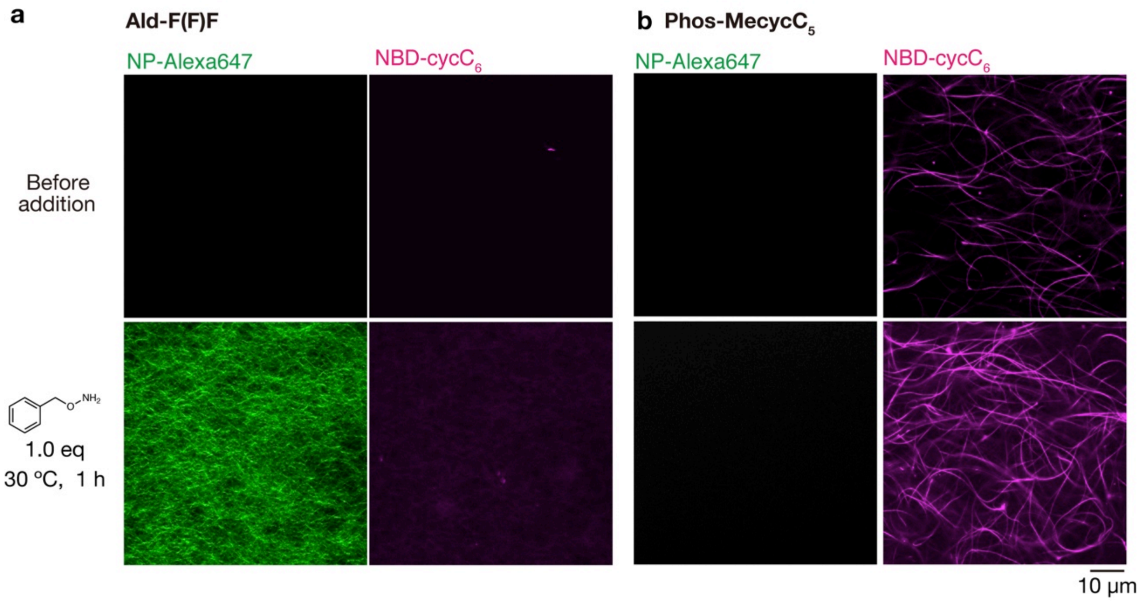

Supplementary Figure. 7. Staining selectivity of fluorescent probes. CLSM imaging of single-component samples ((**a**) **Ald-F(F)F** or (**b**) **Phos-MecycC$_5$**) containing both two fluorescent probes was conducted. Condition: [**Ald-F(F)F**] = 4.3 mM (0.2 wt%), [**Phos-MecycC$_5$**] = 2.4 mM (0.15 wt%), [**NP-Alexa647**] = 4.0 µM, [**NBD-cycC$_6$**] = 4.0 µM, [*O*-benzylhydroxylamine] = 4.3 mM (1.0 eq) in 100 mM MES, pH 6.0.

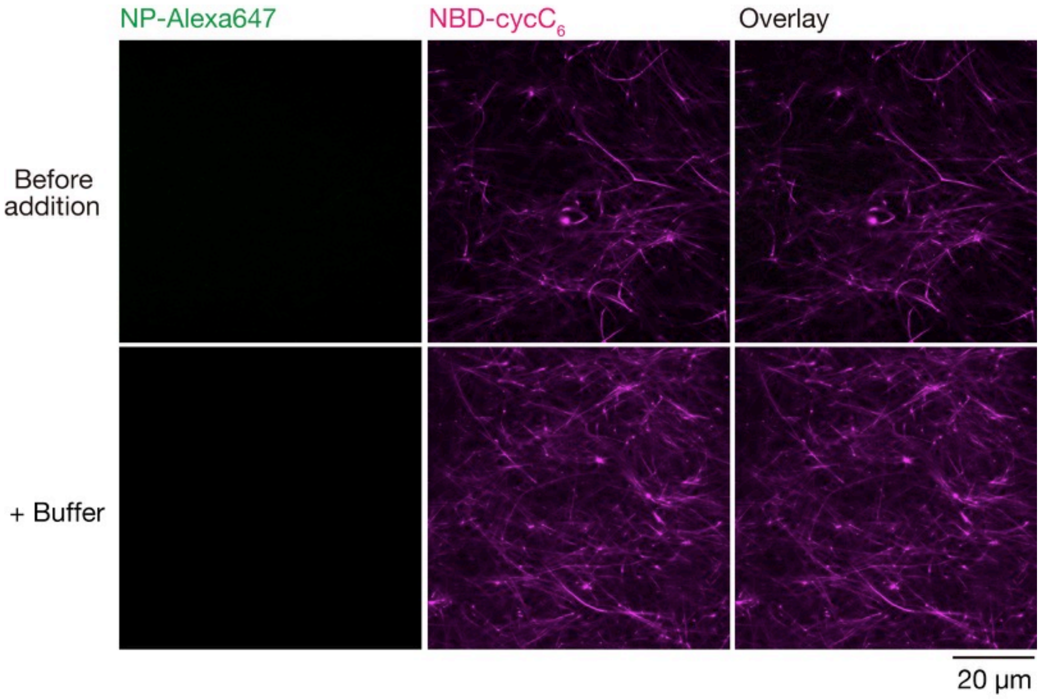

Supplementary Figure 8. CLSM imaging before and after buffer addition to a mixture of **Ald-F(F)F** and **Phos-MecycC$_5$**. Condition: [**Ald-F(F)F**] = 4.3 mM (0.2 wt%), [**Phos-MecycC$_5$**] = 2.4 mM (0.15 wt%), [**NP-Alexa647**] = 4.0 μM, [**NBD-cycC$_6$**] = 4.0 μM in 100 mM MES, pH 6.0.

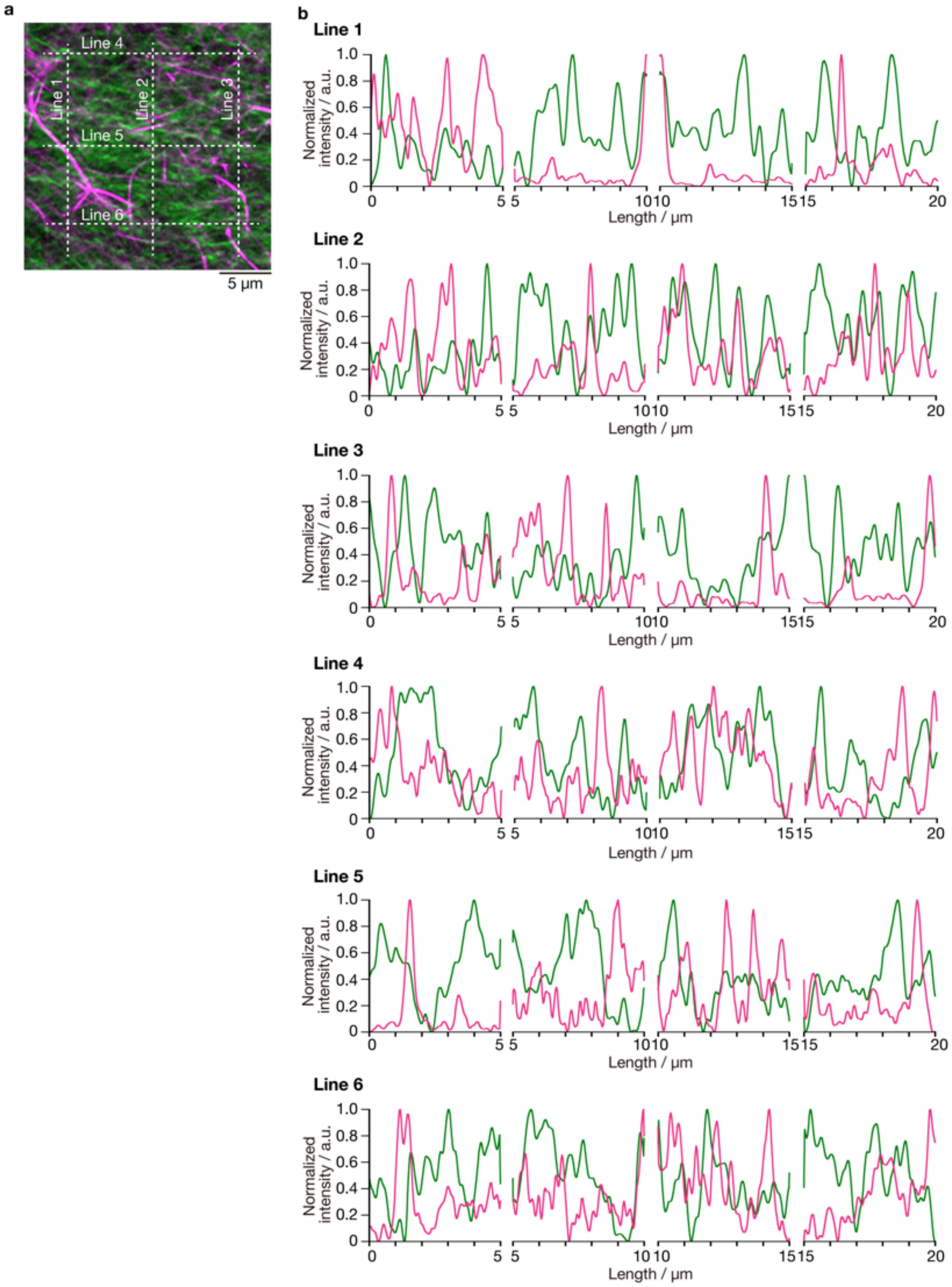

Supplementary Figure 9. (**a**) High-resolution Airyscan CLSM image of the interpenetrated SDN formed by the oxime formation protocol (same as Fig. 3b, bottom). Green: **NP-Alexa647**, magenta: **NBD-cycC$_6$**. (**b**) Line plot analyses along white lines shown in supplementary Fig. 9a. To compare the peak tops of the peptide- and lipid-type nanofibers, the peak intensity was normalized at 5 μm intervals so that the maximum and minimum intensities were set to 1 and 0, respectively.

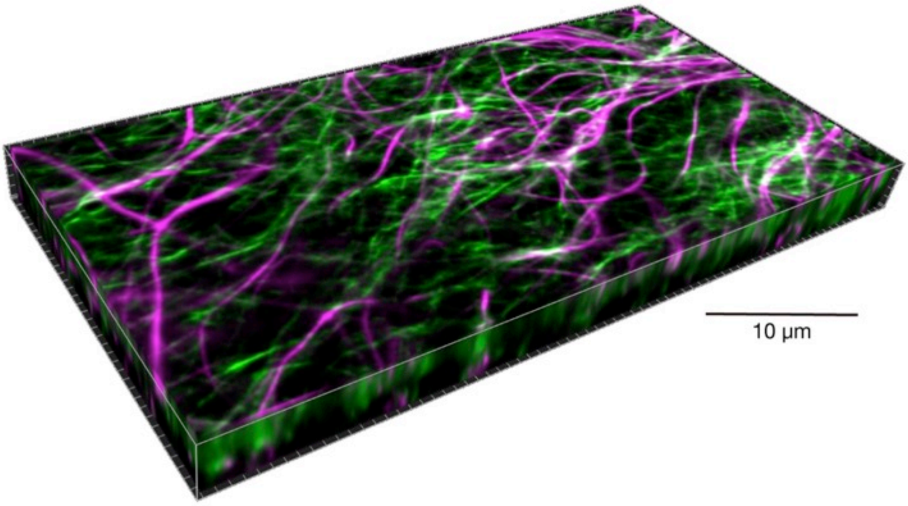

Supplementary Figure 10. 3D z-stacked CLSM imaging of the interpenetrated self-sorting network of **Ald-F(F)F** and **Phos-MecycC$_5$** after treatment of *O*-benzylhydroxylamine. Green: **NP-Alexa647**, magenta: **NBD-cycC$_6$**. Condition: [**Ald-F(F)F**] = 4.3 mM (0.20 wt%), [**Phos-MecycC$_5$**] = 2.4 mM (0.15 wt%), [*O*-benzylhydroxylamine] = 4.3 mM (1.0 eq), [**NP-Alexa647**] = 4.0 μM, [**NBD-cycC$_6$**] = 4.0 μM, 100 mM MES, pH 6.0, 30 ºC.

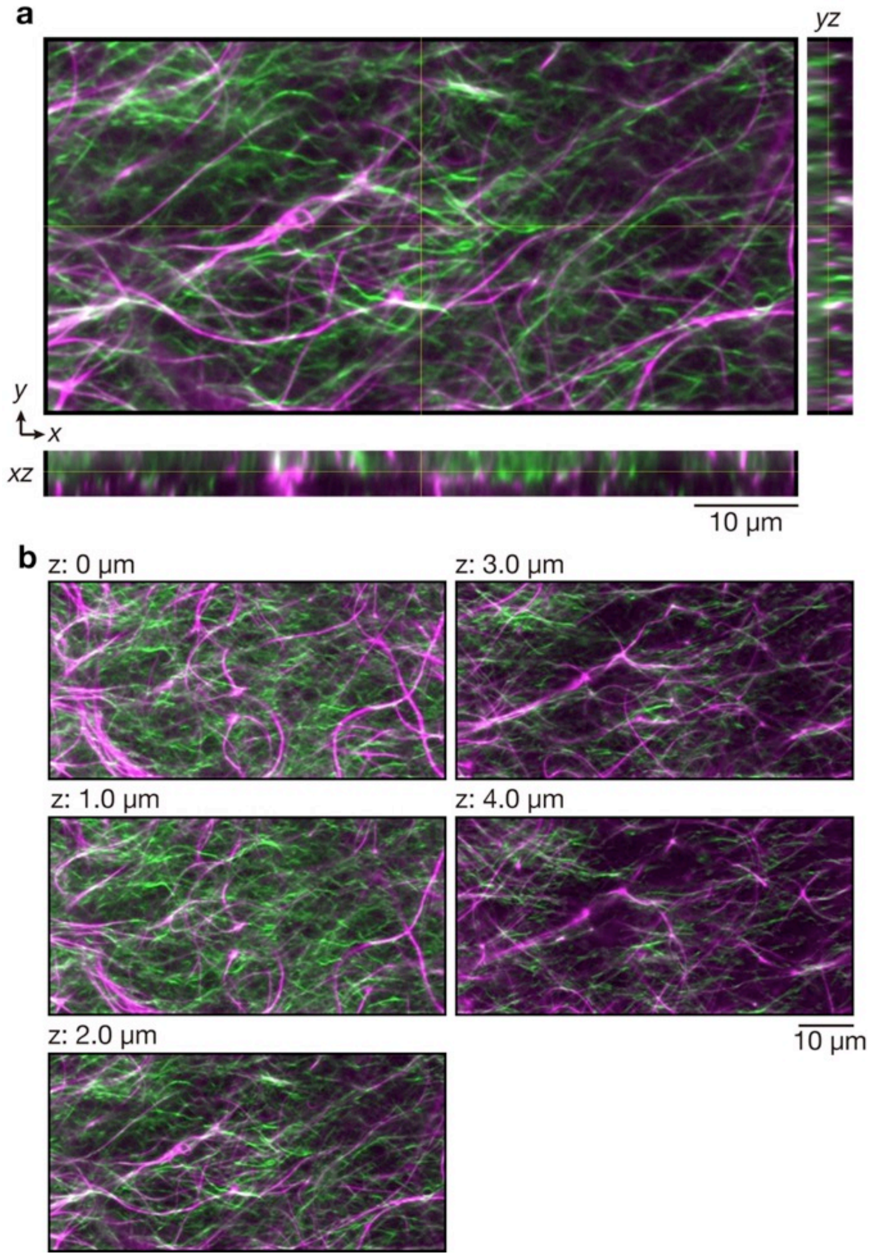

Supplementary Figure 11. (**a**) Orthosteric view and (**b**) z slice images of the interpenetrated self-sorting network of **Ald-F(F)F** and **Phos-MecycC$_5$** after treatment of *O*-benzylhydroxylamine. Green: **NP-Alexa647**, magenta: **NBD-cycC$_6$**. Condition: [**Ald-F(F)F**] = 4.3 mM (0.20 wt%), [**Phos-MecycC$_5$**] = 2.4 mM (0.15 wt%), [*O*-benzylhydroxylamine] = 4.3 mM (1.0 eq), [**NP-Alexa647**] = 4.0 μM, [**NBD-cycC$_6$**] = 4.0 μM, 100 mM MES, pH 6.0, 30 ºC.

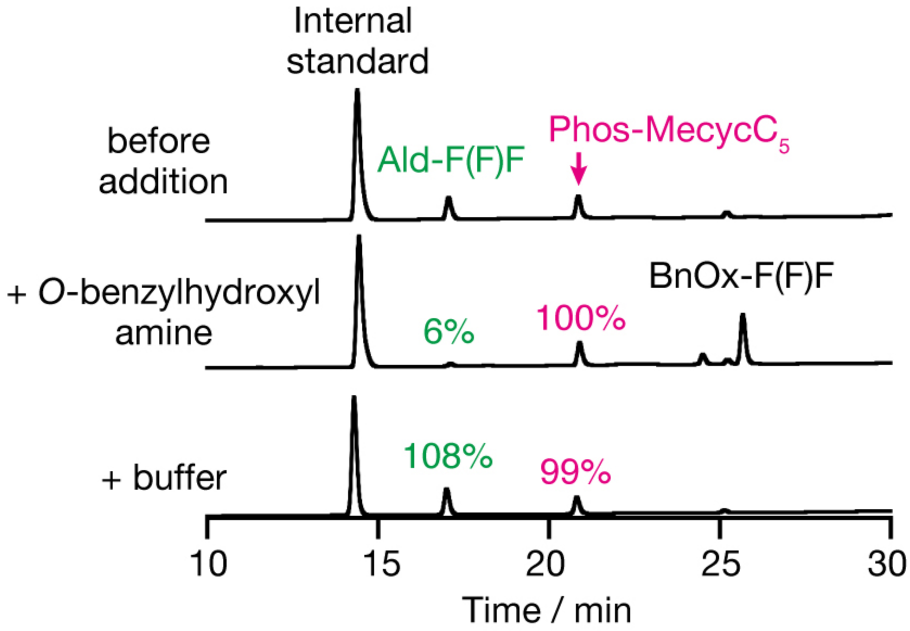

Supplementary Figure 12. HPLC analysis (top) before and after treatment of (middle) *O*-benzylhydroxylamine or (bottom) buffer. Internal standard: fluorescein. The residual ratio of **Ald-F(F)F** was determined by comparing the integral ratio before *O*-benzylhydroxylamine addition as 100%. Condition: [**Ald-F(F)F**] = 4.3 mM (0.2 wt%), [**Phos-MecycC$_5$**] = 2.4 mM (0.15 wt%), [*O*-benzylhydroxylamine] = 4.3 mM (1.0 eq) in 100 mM MES, pH 6.0.

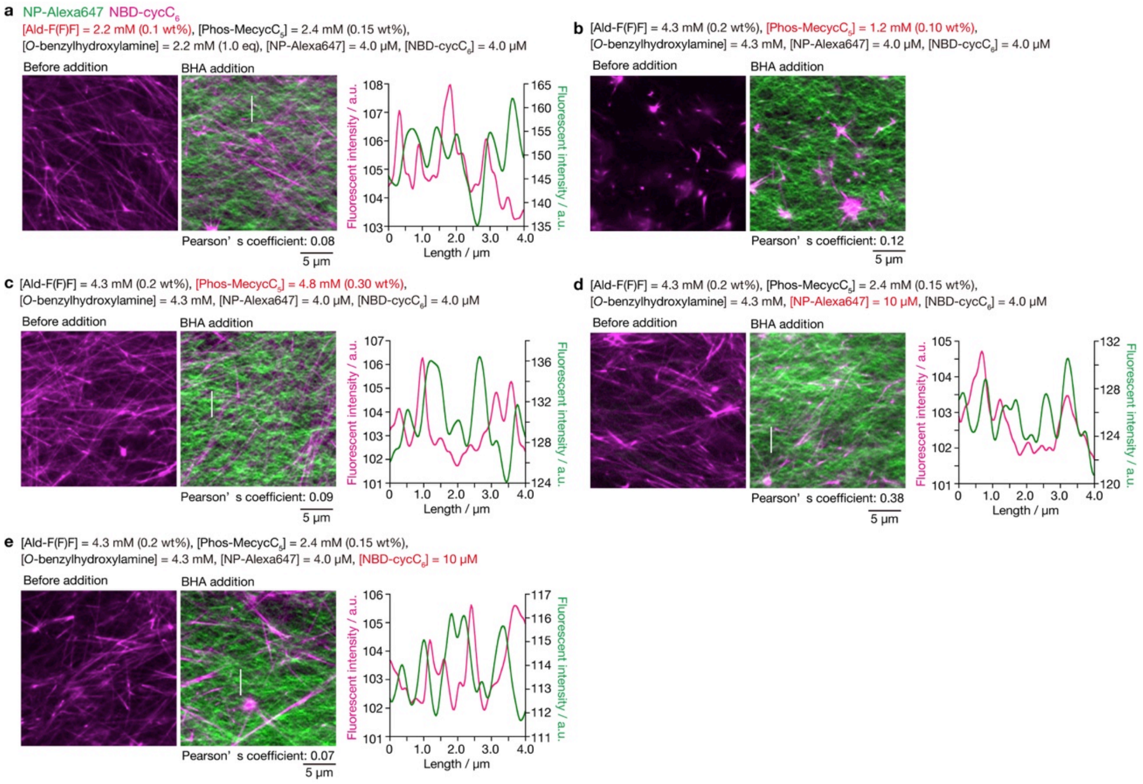

Supplementary Figure 13. CLSM imaging of the mixture of **Ald-F(F)F** and **Phos-MecycC$_5$** (left) before and (middle) after treatment of *O*-benzylhydroxylamine under various conditions. Spherical aggregates mainly formed when using lower amount of **Phos-MecycC$_5$** (1.2 mM) (supplementary Fig. 13b). (Right) Line plot analysis along white lines shown in the middle images. Green: **NP-Alexa647**, magenta: **NBD-cycC$_6$**. Condition: 100 mM MES, pH 6.0, 30 ºC.

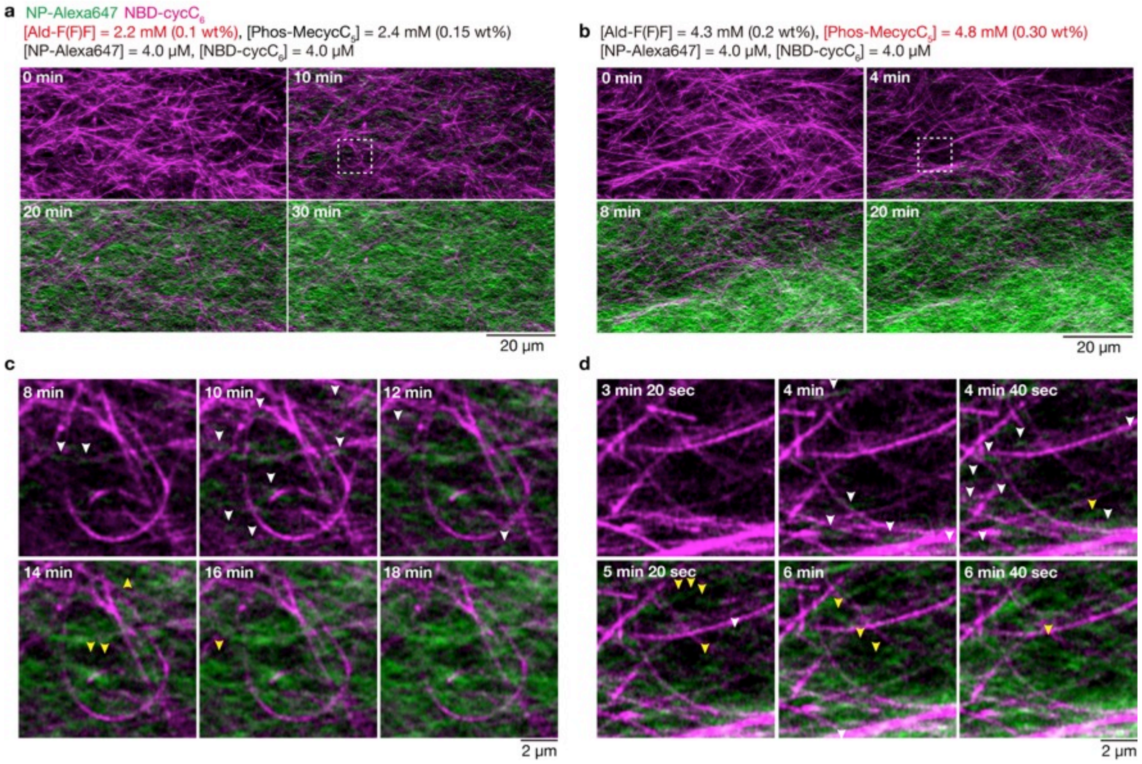

Supplementary Figure 14. (**a**,**b**) Time lapse imaging of the formation of the interpenetrated SDN through the oxime formation. (**c**,**d**) Magnified view of the formation of peptide-type nanofibers on the surface of lipid-type nanofibers (highlighted by white arrows) and in the water layer (highlighted by yellow arrows). The white square region in supplementary Fig. 14a and 14b were magnified. Green: **NP-Alexa647**, magenta: **NBD-cycC$_6$**. Condition: [**Ald-F(F)F**] = (**a**,**c**) 2.2 or (**b**,**d**) 4.3 mM (0.10 or 0.20 wt%), [**Phos-MecycC$_5$**] = (**a**,**c**) 2.4 or (**b**,**d**) 4.8 mM (0.15 or 0.30 wt%), [*O*-benzylhydroxylamine] = (**a**,**c**) 2.2 or (**b**,**d**) 4.3 mM (1.0 eq), [**NP-Alexa647**] = 4.0 μM, [**NBD-cycC$_6$**] = 4.0 μM, 100 mM MES, pH 6.0, 30 ºC.

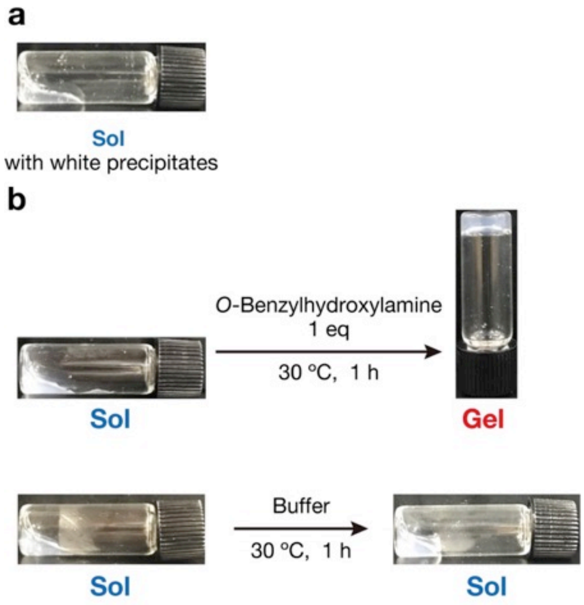

Supplementary Figure 15. (**a**) Macroscopic observation of the mixture of **BnOx-F(F)F** and **Phos-MecycC$_5$**. Condition: [**BnOx-F(F)F**] = 4.3 mM (0.25 wt%) [**Phos-MecycC$_5$**] = 2.4 mM (0.15 wt%) in 100 mM MES, pH 6.0. (**b**) Macroscopic sol-gel transition upon addition of (top) *O*-benzylhydroxylamine and (bottom) buffer to a mixture of **Ald-F(F)F** and **Phos-MecycC$_5$**. Conditions: [**Ald-F(F)F**] = 4.3 mM (0.2 wt%) [**Phos-MecycC$_5$**] = 2.4 mM (0.15 wt%), [*O*-benzylhydroxylamine] = 4.3 mM (1.0 eq) in 100 mM MES, pH 6.0.

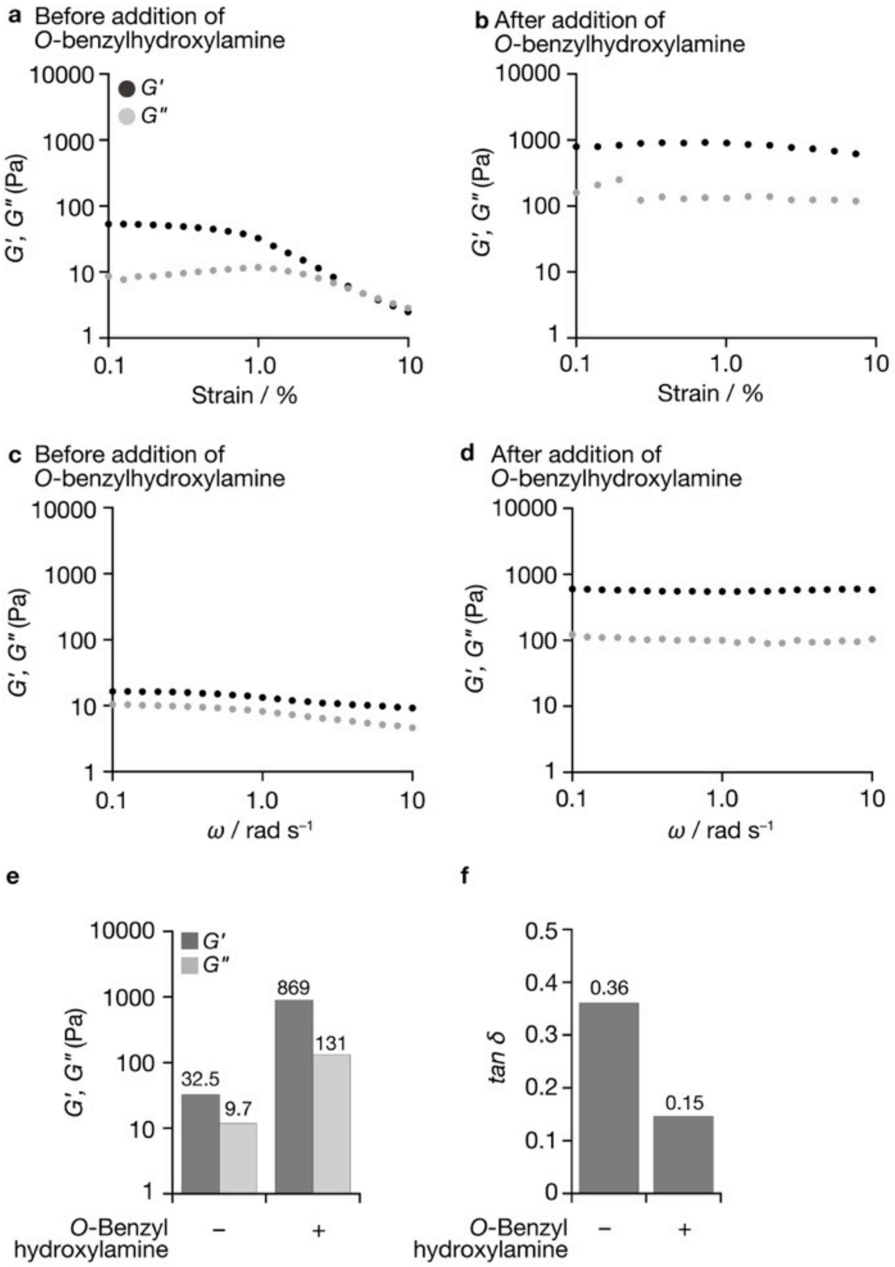

Supplementary Figure 16. (**a**,**b**) Strain and (**c**,**d**) frequency sweep rheological properties of **Ald-F(F)F**/**Phos-MecycC$_5$** solution (**a**,**c**) before and (**b**,**d**) after addition of *O*-benzylhydroxylamine. Frequency for strain sweep: 10 rad/s. Strain amplitude for frequency sweep: 1%. *G′*: storage shear modulus, *G″*: loss shear modulus. (**e**) *G′*, *G″* and (**f**) tan δ values of the **Ald-F(F)F**/**Phos-MecycC$_5$** before and after addition of *O*-benzylhydroxylamine. Frequency: 10 rad/s, strain amplitude: 1%. Condition: [**Ald-F(F)F**] = 4.3 mM (0.2 wt%), [**Phos-MecycC$_5$**] = 2.4 mM (0.15 wt%), [*O*-benzylhydroxylamine] = 4.3 mM (1.0 eq) in 100 mM MES, pH 6.0.

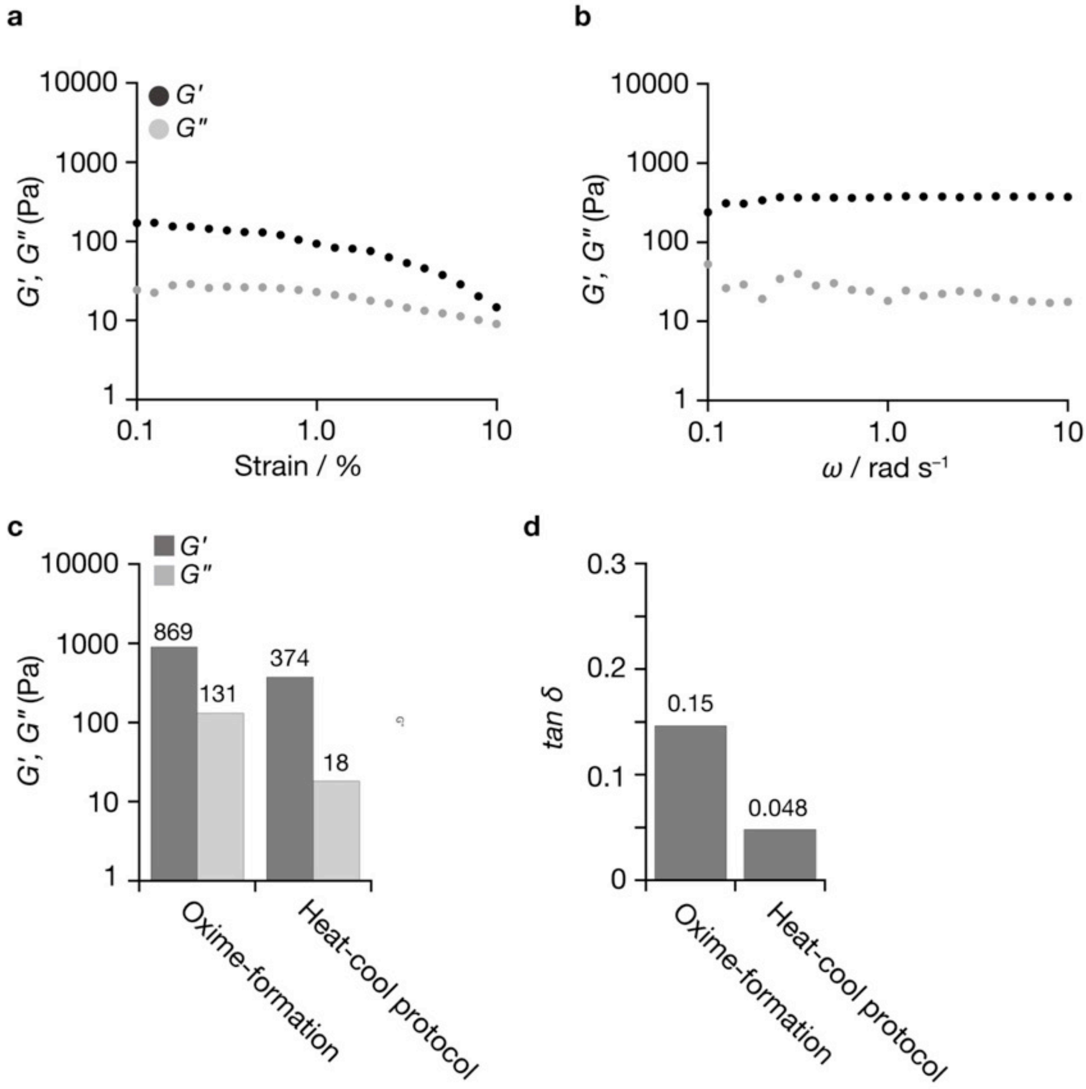

Supplementary Figure 17. (**a**) Strain and (**b**) frequency sweep rheological properties of the suspension of **BnOx-F(F)F**/**Phos-MecycC$_5$**. Frequency for strain sweep: 10 rad/s. Strain amplitude for frequency sweep: 1%. *G'*: storage shear modulus, *G"*: loss shear modulus. (**c**) *G'*, *G"* and (**d**) tan δ values of the hydrogel prepared by *O*-benzylhydroxylamine addition to **Ald-F(F)F**/**Phos-MecycC$_5$** (the oxime-formation protocol) and the suspension of **BnOx-F(F)F**/**Phos-MecycC$_5$** (the heat-cool protocol). Frequency: 10 rad/s, strain amplitude: 1%. Condition: [**Ald-F(F)F**] = 4.3 mM (0.2 wt%), [**BnOx-F(F)F**] = 4.3 mM (0.25 wt%), [**Phos-MecycC$_5$**] = 2.4 mM (0.15 wt%), [*O*-benzylhydroxylamine] = 4.3 mM (1.0 eq) in 100 mM MES, pH 6.0.

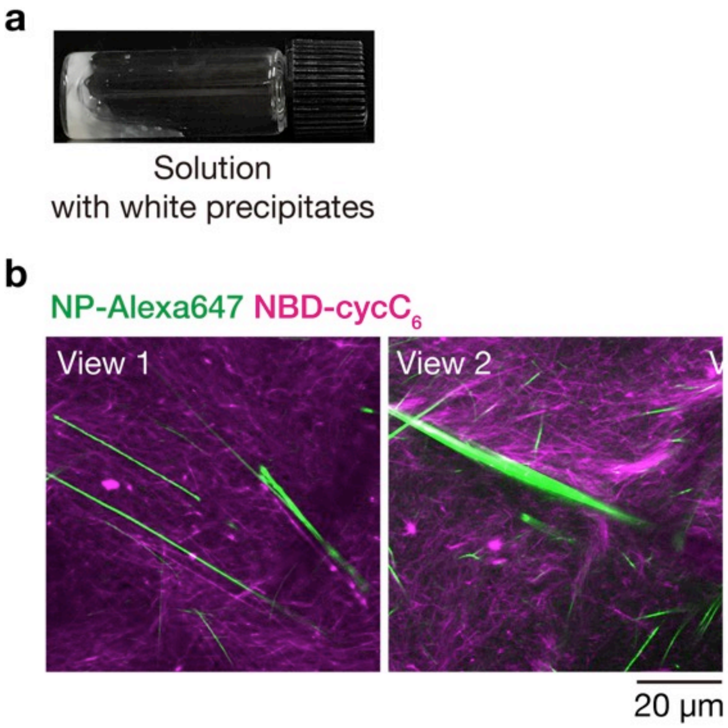

Supplementary Figure 18. (**a**) Macroscopic observation of the mixture of **BnOx-F(F)F** and **Phos-MecycC$_5$**, which was prepared by mixing hot solutions of **BnOx-F(F)F** and **Phos-MecycC$_5$**. (**b**) CLSM imaging of the mixture of **BnOx-F(F)F**, **Phos-MecycC$_5$**, **NP-Alexa647**, and **NBD-cycC$_6$**. Two different views were shown. These CLSM images showed that **BnOx-F(F)F** and **Phos-MecycC$_5$** nanofibers were self-sorted but formed the heterogeneous mixture. Condition: [**BnOx-F(F)F**] = 4.3 mM (0.25 wt%) [**Phos-MecycC$_5$**] = 2.4 mM (0.15 wt%), [**NP-Alexa647**] = 4.0 μM, [**NBD-cycC$_6$**] =4.0 μM in 100 mM MES, pH 6.0.

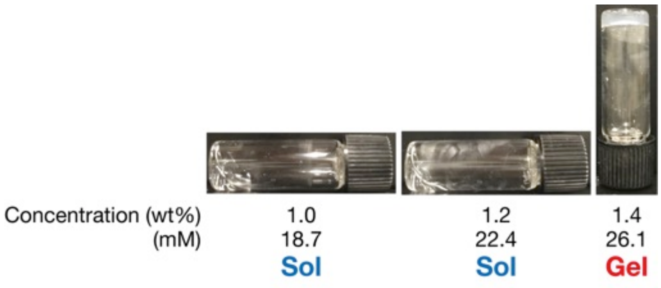

Supplementary Figure 19. Determination of critical gelation concentration of **CaOx-F(F)F**. Condition: 100 mM MES, pH 6.0.

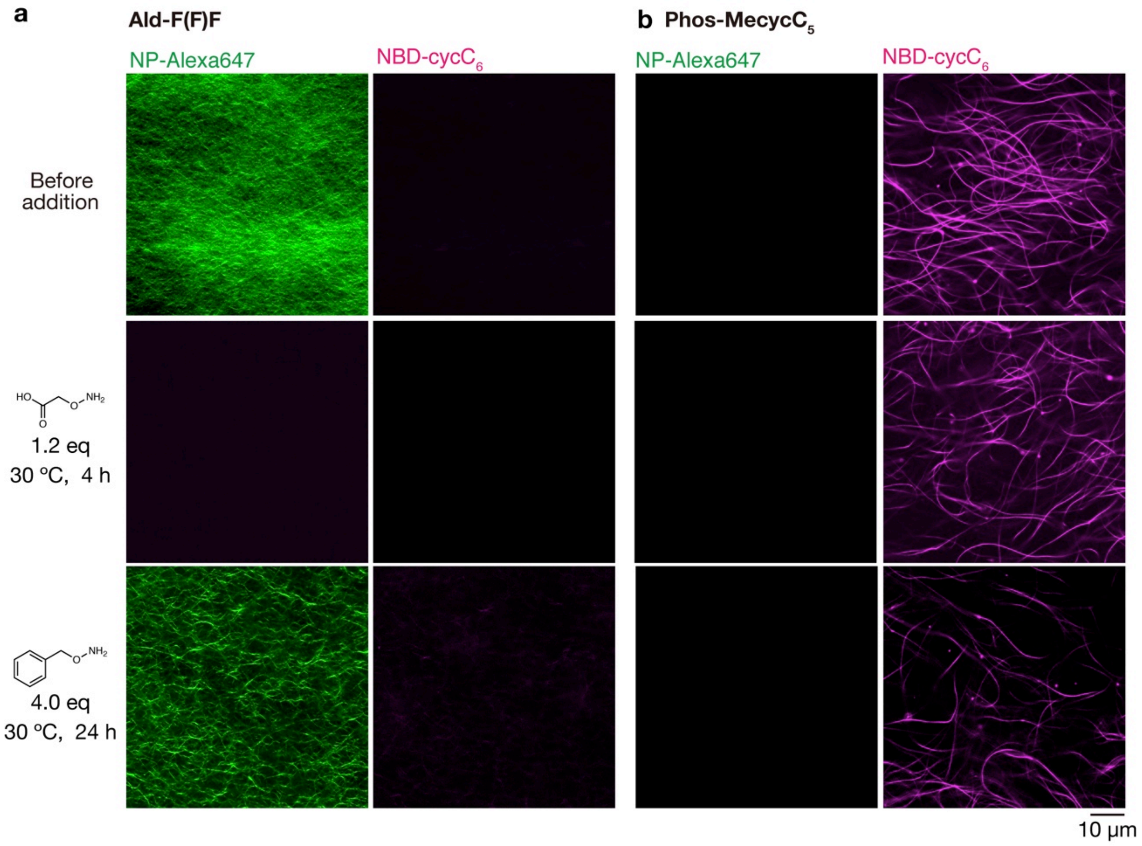

Supplementary Figure 20. Staining selectivity of fluorescent probes. CLSM imaging of single-component samples ((**a**) **Ald-F(F)F** or (**b**) **Phos-MecycC$_5$**) containing both two fluorescent probes were conducted. Condition: (**a**) [**Ald-F(F)F**] = 17.3 mM (0.80 wt%), (**b**) [**Phos-MecycC$_5$**] = 2.4 mM (0.15 wt%), (**a**,**b**) [**NP-Alexa647**] = 4.0 μM, [**NBD-cycC$_6$**] = 4.0 μM, [carboxymethoxylamine] = 21 mM (1.2 eq), [*O*-benzylhydroxylamine] = 69 mM (4.0 eq) in 100 mM MES, pH 6.0.

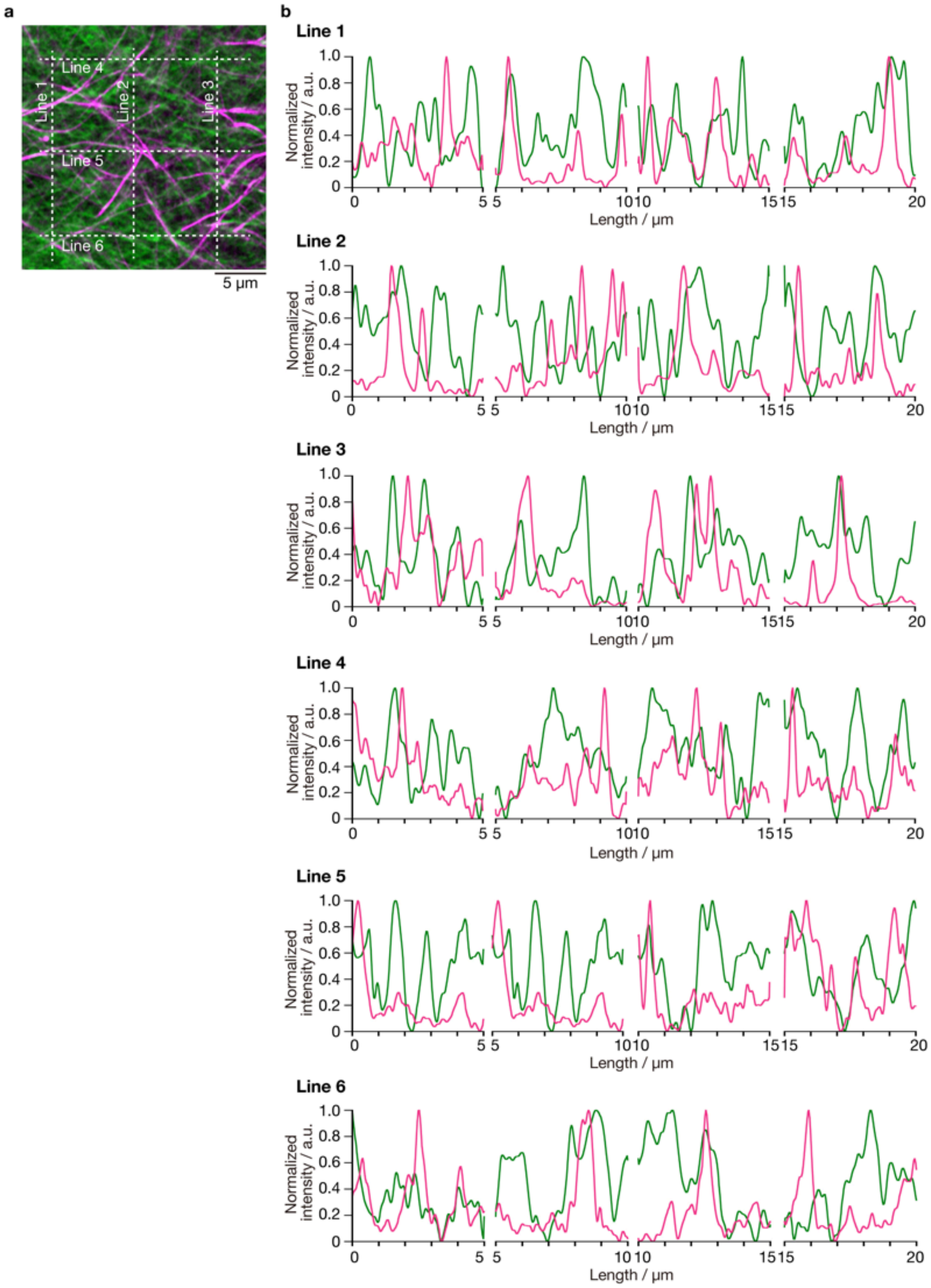

Supplementary Figure 21. (**a**) High-resolution Airyscan CLSM image of the interpenetrated SDN of **Ald-F(F)F** and **Phos-MecycC$_5$** (same as Fig. 5b, top). Green: **NP-Alexa647**, magenta: **NBD-cycC$_6$**. (**b**) Line plot analyses along white lines shown in supplementary Fig. 21a. To compare the peak tops of the peptide- and lipid-type nanofibers, the peak intensity was normalized at 5 µm intervals so that the maximum and minimum intensities were set to 1 and 0, respectively.

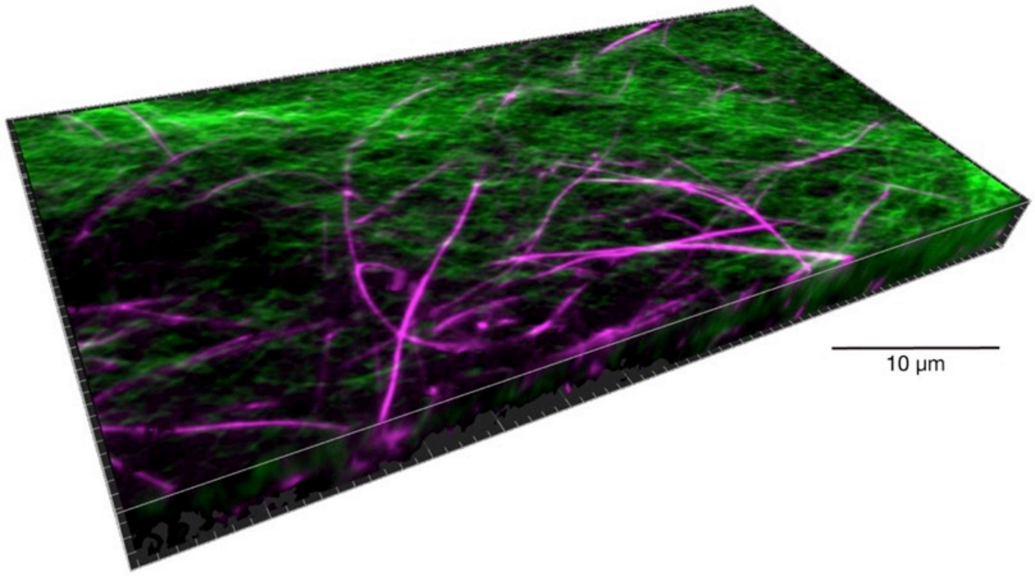

Supplementary Figure 22. 3D z-stacked CLSM imaging of the interpenetrated self-sorting network of **Ald-F(F)F** and **Phos-MecycC$_5$**. Green: **NP-Alexa647**, magenta: **NBD-cycC$_6$**. Condition: [**Ald-F(F)F**] = 17.3 mM (0.80 wt%), [**Phos-MecycC$_5$**] = 2.4 mM (0.15 wt%), [**NP-Alexa647**] = 4.0 μM, [**NBD-cycC$_6$**] = 4.0 μM in 100 mM MES, pH 6.0.

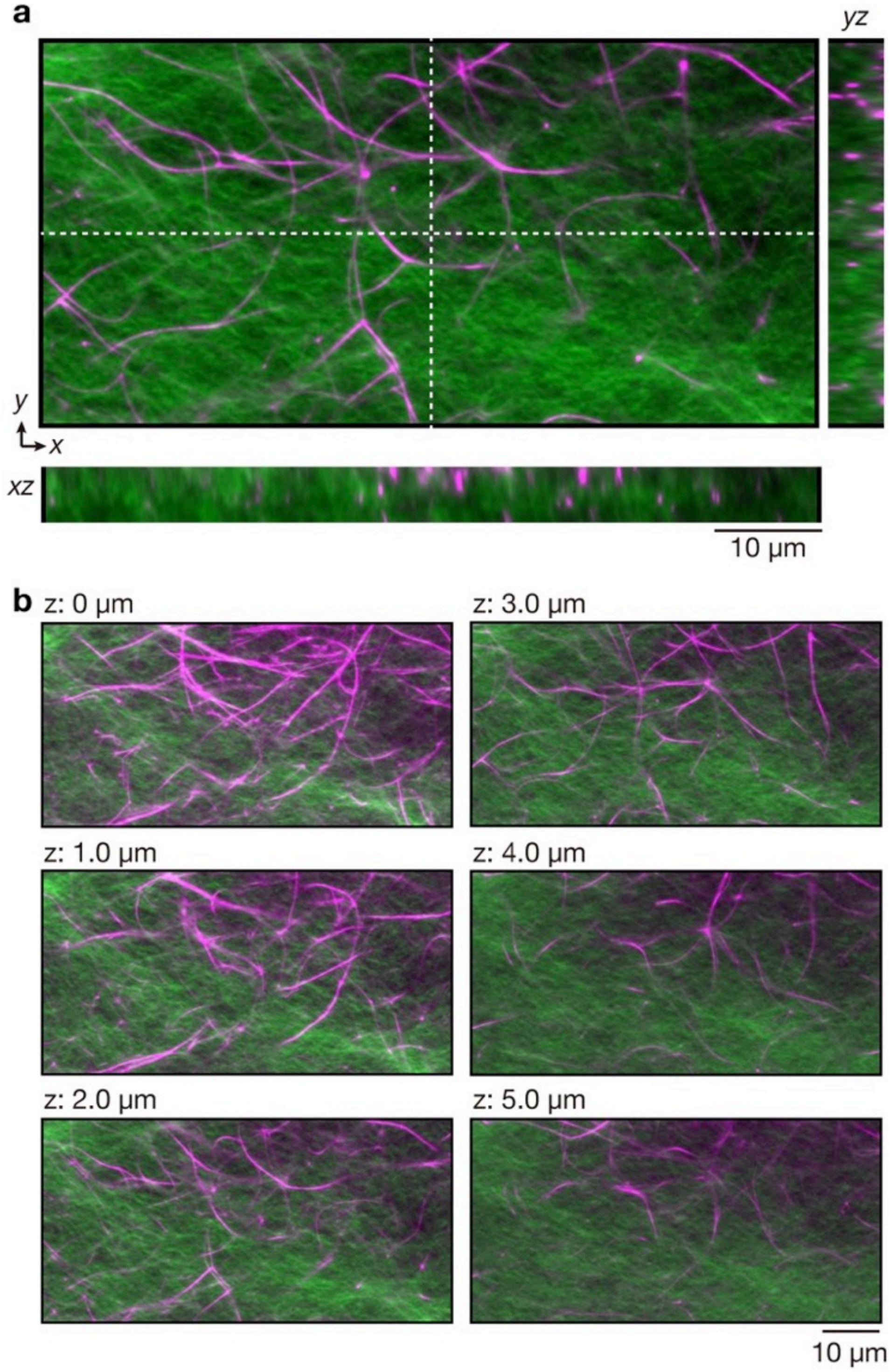

Supplementary Figure 23. (**a**) Orthosteric view and (**b**) z slice images of the interpenetrated self-sorting network of **Ald-F(F)F** and **Phos-MecycC$_5$**. Green: **NP-Alexa647**, magenta: **NBD-cycC$_6$**. Condition: [**Ald-F(F)F**] = 17.3 mM (0.80 wt%), [**Phos-MecycC$_5$**] = 2.4 mM (0.15 wt%), [**NP-Alexa647**] = 4.0 μM, [**NBD-cycC$_6$**] = 4.0 μM in 100 mM MES, pH 6.0.

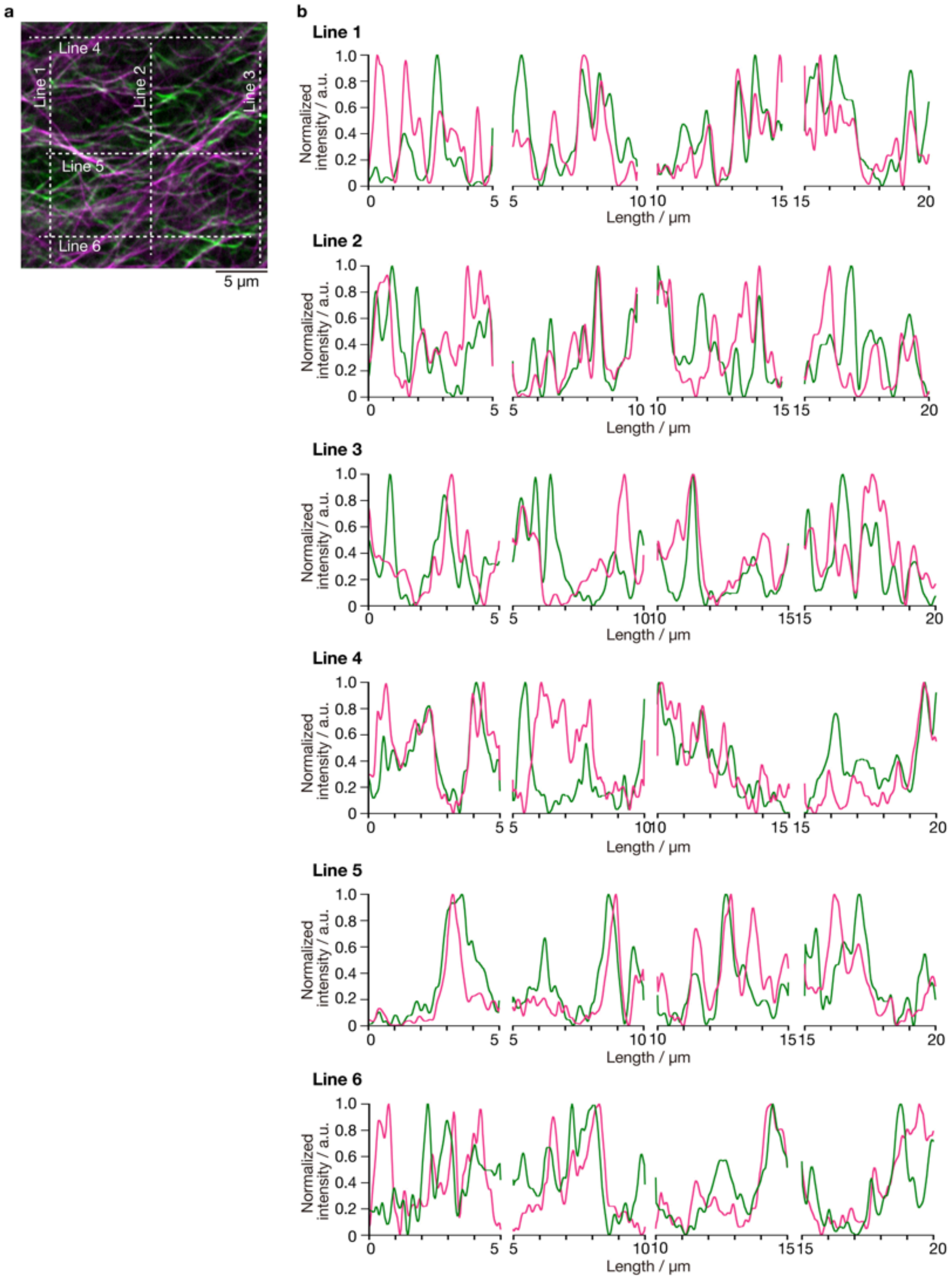

Supplementary Figure 24. (**a**) High-resolution Airyscan CLSM image of the parallel SDN (same as Fig. 5b, bottom). Green: **NP-Alexa647**, magenta: **NBD-cycC$_6$**. (**b**) Line plot analyses along white lines shown in supplementary Fig. 24a. To compare the peak tops of the peptide- and lipid-type nanofibers, the peak intensity was normalized at 5 μm intervals so that the maximum and minimum intensities were set to 1 and 0, respectively.

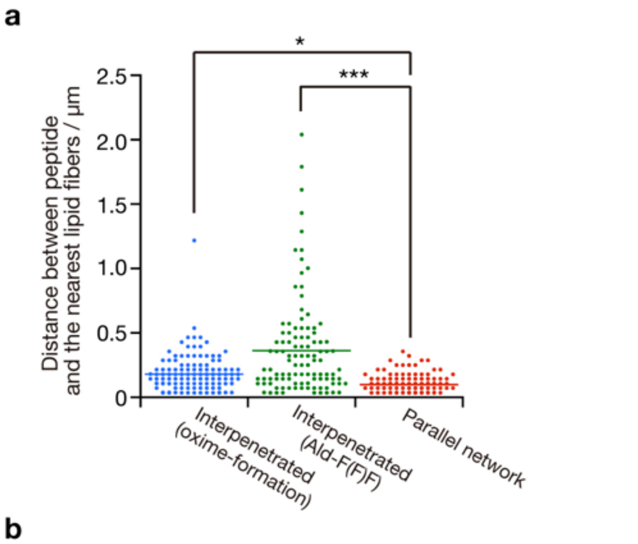

b

	Interpenetrated (oxime-formation)	Interpenetrated (Ald-F(F)F)	Parallel
Peak distance (nm)	180 ± 150	360 ± 380	98 ± 80

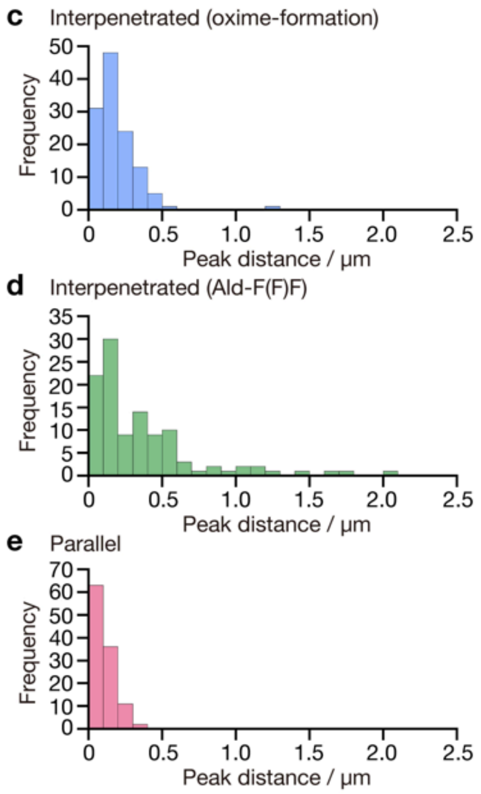

Supplementary Figure 25. Quantitative analysis of peak-top distances between peptide- and the nearest lipid-type nanofibers. (**a**) Dot plot analysis. (**b**) Average peak-top distances. Histograms of the peak-top distance in (**c**) the interpenetrated network formed by the oxime-formation protocol (*n* = 122, supplementary Fig. 9), (**d**) the interpenetrated network of **Ald-F(F)F**/**Phos-MecycC$_5$** (*n* = 109, supplementary Fig. 21), and (**e**) the parallel network (*n* = 111, supplementary Fig. 24). The quantitative analysis suggested that the peptide- and lipid-type nanofibers located mainly within a distance of 100 nm in the parallel network (**e**). In contrast, they mainly located over 100 nm in the interpenetrated network (**d**). The interpenetrated network formed by the oxime-formation protocol (**c**) showed the intermediate peak-top distance and distribution, as indicated by the time-lapse imaging (the mixture of the interpenetrated and parallel networks). Single and triple asterisks (*, ***) denote significant differences from the group of the parallel SDN (*: $P = 0.0175$, ***: $P < 0.001$, one way analysis of variance with Dunnett's *post huc* test). F(2/342) = 35.35.

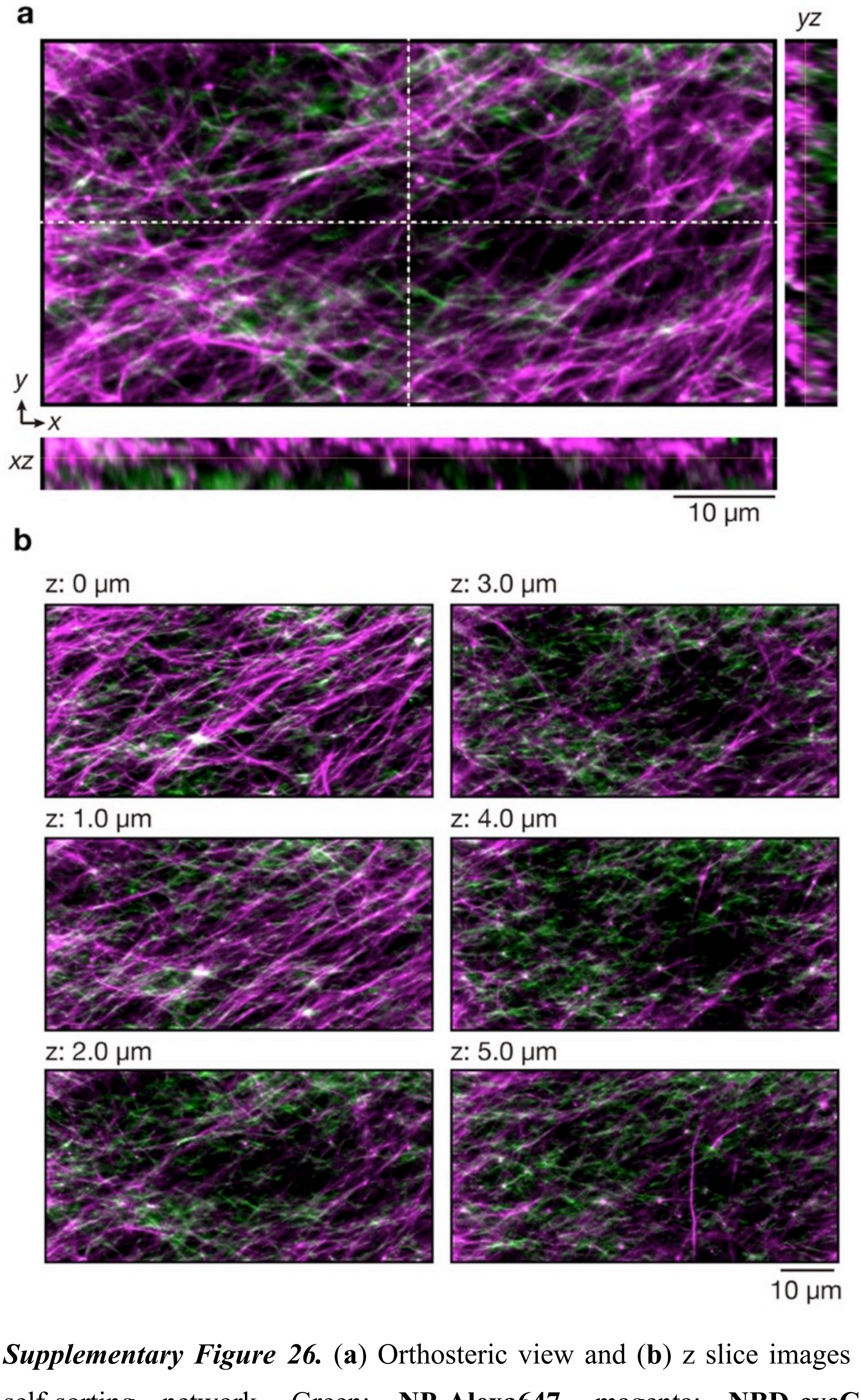

Supplementary Figure 26. (**a**) Orthosteric view and (**b**) z slice images of the parallel self-sorting network. Green: **NP-Alexa647**, magenta: **NBD-cycC$_6$**. Condition: [**Ald-F(F)F**] = 17.3 mM (0.80 wt%), [**Phos-MecycC$_5$**] = 2.4 mM (0.15 wt%), [**NP-Alexa647**] = 4.0 μM, [**NBD-cycC$_6$**] = 4.0 μM, [carboxymethoxylamine] = 21 mM (1.2 eq), [*O*-benzylhydroxylamine] = 69 mM (4.0 eq) in 100 mM MES, pH 6.0.

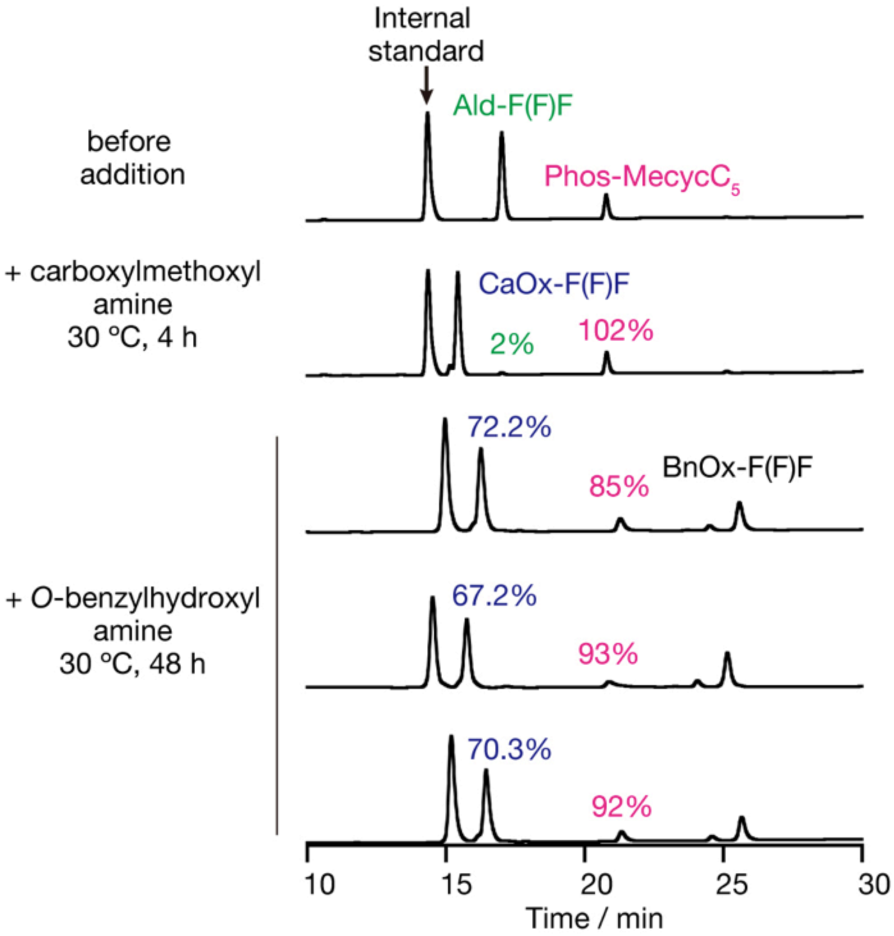

Supplementary Figure 27. HPLC analyses of the oxime exchange process. The average oxime exchange rate was determined to be 30 ± 2% (the mean ± standard deviation, n = 3). Internal standard: fluorescein. The residual ratio of **Ald-F(F)F** and **CaOx-F(F)F** were determined by comparing the integral ratio of **Ald-F(F)F** (the first row) and **CaOx-F(F)F** (the second row) as 100%, respectively. Condition: [**Ald-F(F)F**] = 17.3 mM (0.80 wt%), [**Phos-MecycC$_5$**] = 2.4 mM (0.15 wt%), [carboxymethoxylamine] = 21 mM (1.2 eq), [*O*-benzylhydroxylamine] = 69 mM (4.0 eq) in 100 mM MES, pH 6.0.

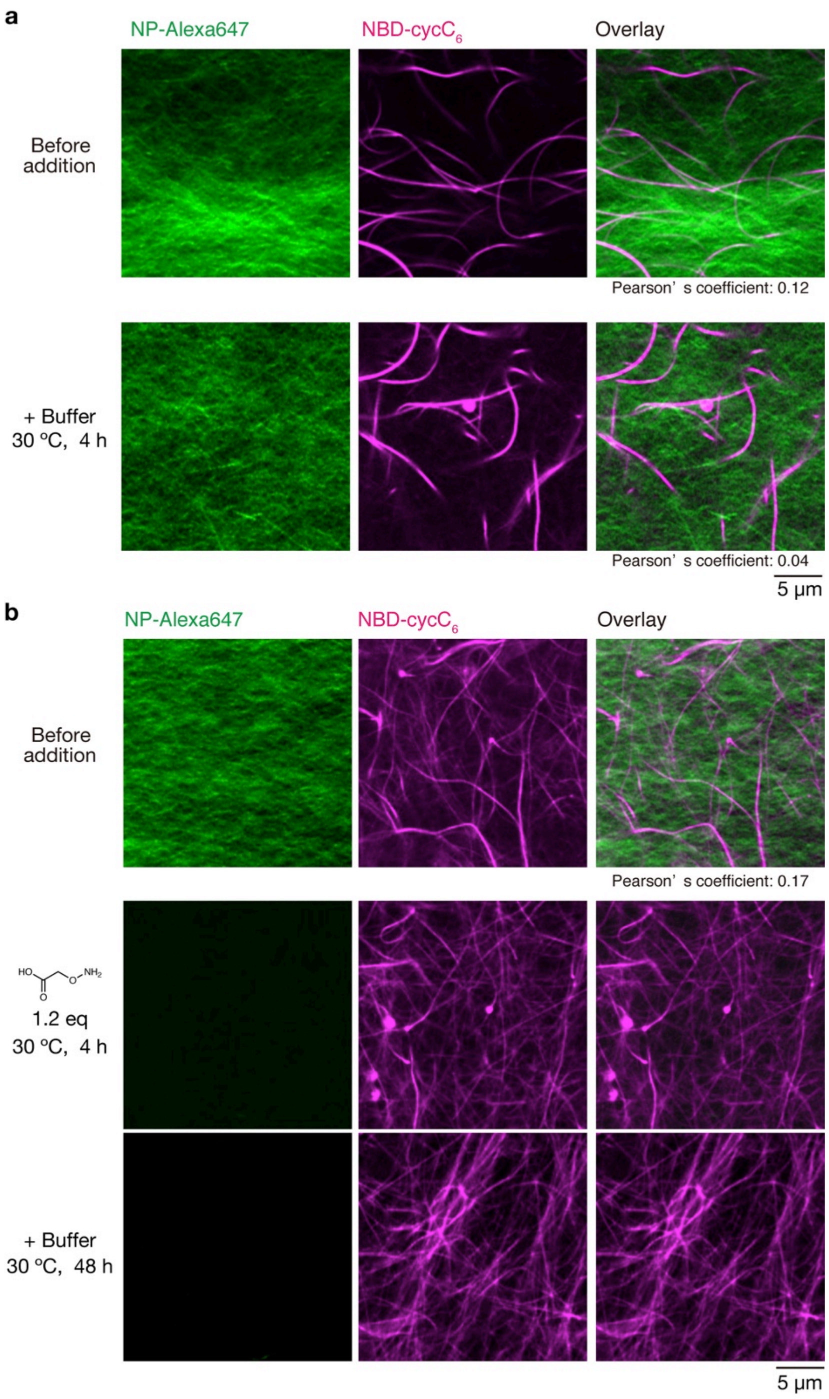

Supplementary Figure 28. CLSM imaging before and after addition of buffer to (**a**) the hydrogel of **Ald-F(F)F** and **Phos-MecycC$_5$** and (**b**) the solution of **CaOx-F(F)F** and **Phos-MecycC$_5$**. Condition: [**Ald-F(F)F**] = 17.3 mM (0.80 wt%), [**Phos-MecycC$_5$**] = 2.4 mM (0.15 wt%), [**NP-Alexa647**] = 4.0 μM, [**NBD-cycC$_6$**] = 4.0 μM, [carboxymethoxylamine] = 21 mM (1.2 eq), [*O*-benzylhydroxylamine] = 69 mM (4.0 eq) in 100 mM MES, pH 6.0.

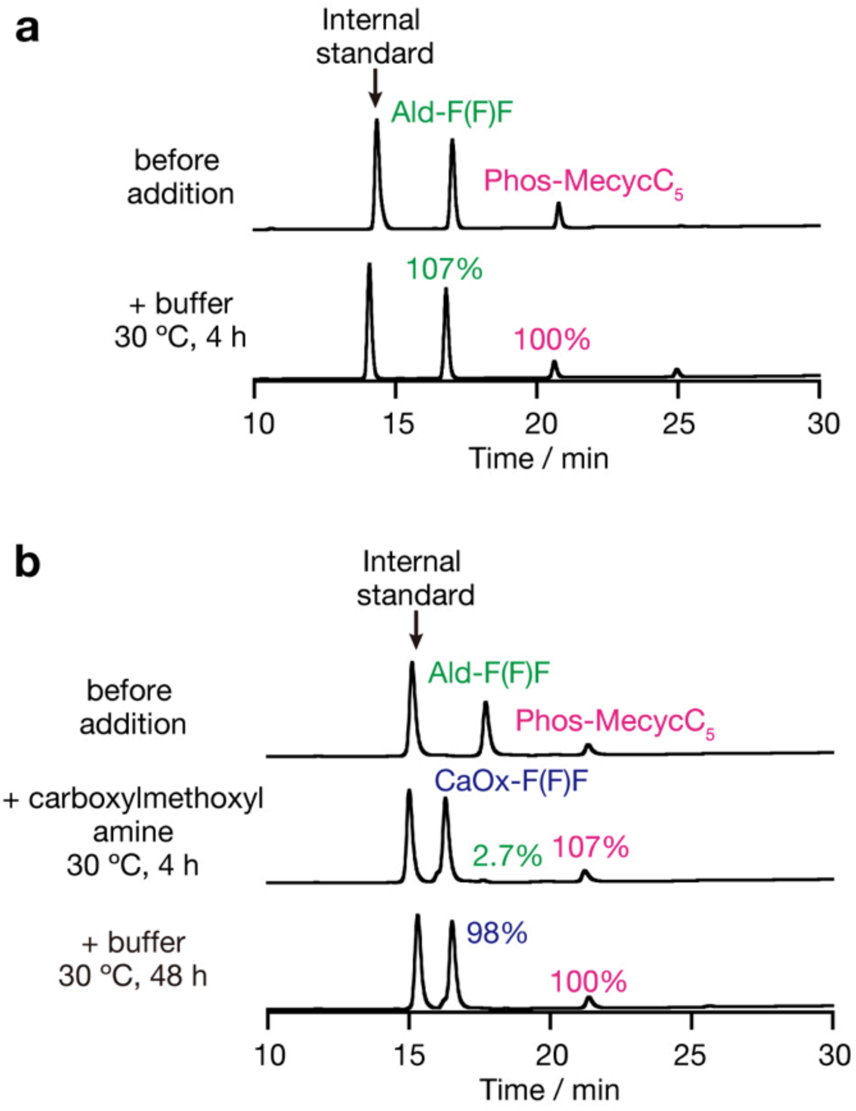

Supplementary Figure 29. HPLC analyses of buffer control experiments. Instead of solutions of (**a**) carboxymethoxylamine and (**b**) *O*-benzylhydroxylamine, the same amount of buffer was added. The residual ratio of **Ald-F(F)F** and **CaOx-F(F)F** were determined by comparing the integral ratio of **Ald-F(F)F** (the first row) and **CaOx-F(F)F** (the second row) as 100%, respectively. Condition: [**Ald-F(F)F**] = 17.3 mM (0.80 wt%), [**Phos-MecycC$_5$**] = 2.4 mM (0.15 wt%), [carboxymethoxylamine] = 21 mM (1.2 eq) in 100 mM MES, pH 6.0.

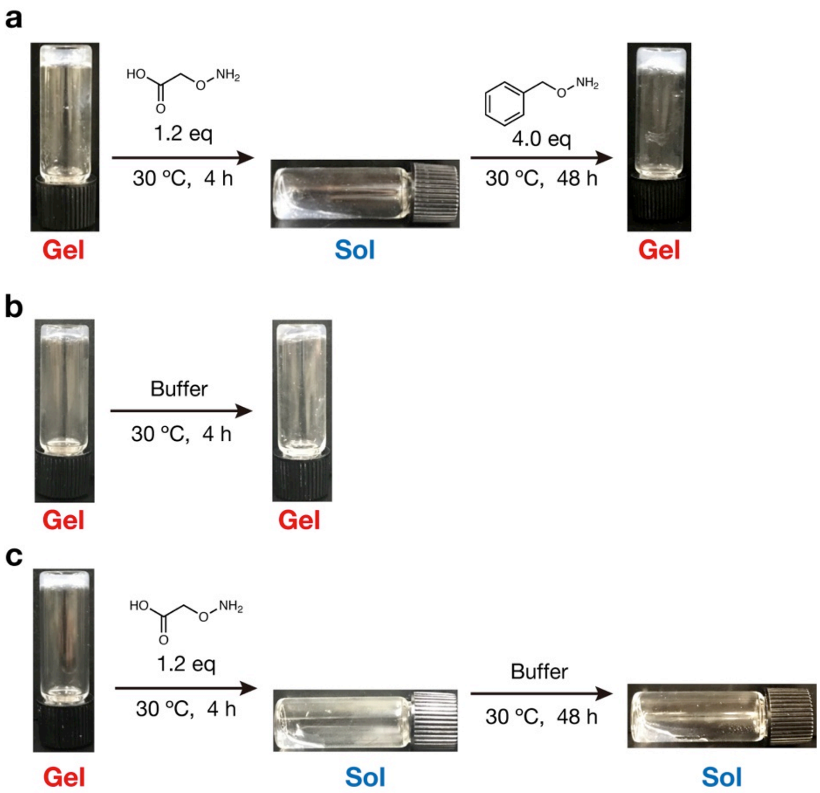

Supplementary Figure 30. Macroscopic gel-sol-gel transition upon sequential addition of (**a**) carboxymethoxylamine and *O*-benzylhydroxylamine, (**b**) buffer, and (**c**) carboxymethoxylamine and buffer to the hydrogel of **Ald-F(F)F** and **Phos-MecycC$_5$**. Condition: [**Ald-F(F)F**] = 17.3 mM (0.80 wt%), [**Phos-MecycC$_5$**] = 2.4 mM (0.15 wt%), [carboxymethoxylamine] = 21 mM (1.2 eq), [*O*-benzylhydroxylamine] = 69 mM (4.0 eq) in 100 mM MES, pH 6.0.

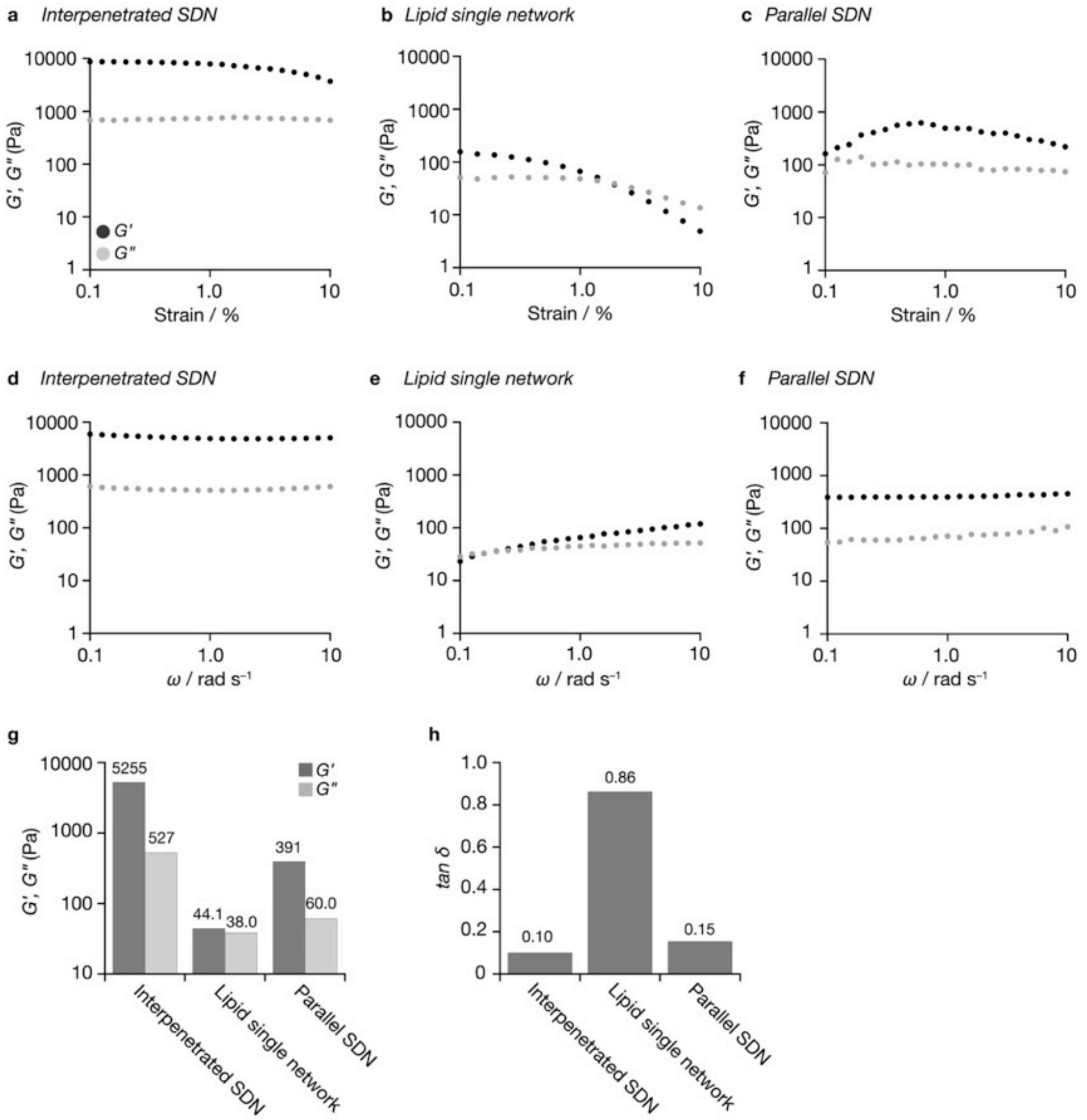

Supplementary Figure 31. (**a**–**c**) Strain and (**d**–**f**) frequency sweep rheological properties of (**a**,**d**) the interpenetrated SDN hydrogel, (**b**,**e**) the single network, and (**c**,**f**) the parallel SDN hydrogel. Frequency for strain sweep: 10 rad/s. Strain amplitude for frequency sweep: (**e**) 0.3 or (**d**,**f**) 1.0%. *G′*: storage shear modulus, *G″*: loss shear modulus. (**g**) *G′*, *G″*, and (**h**) tan δ values of the interpenetrated SDN, single network, and parallel SDN. Frequency: 0.3 rad/s, strain amplitude: 0.3 or 1.0%. Condition: [**Ald-F(F)F**] = 17.3 mM (0.80 wt%), [**Phos-MecycC$_5$**] = 2.4 mM (0.15 wt%), [carboxymethoxylamine] = 21 mM (1.2 eq), [*O*-benzylhydroxylamine] = 69 mM (4.0 eq) in 100 mM MES, pH 6.0.

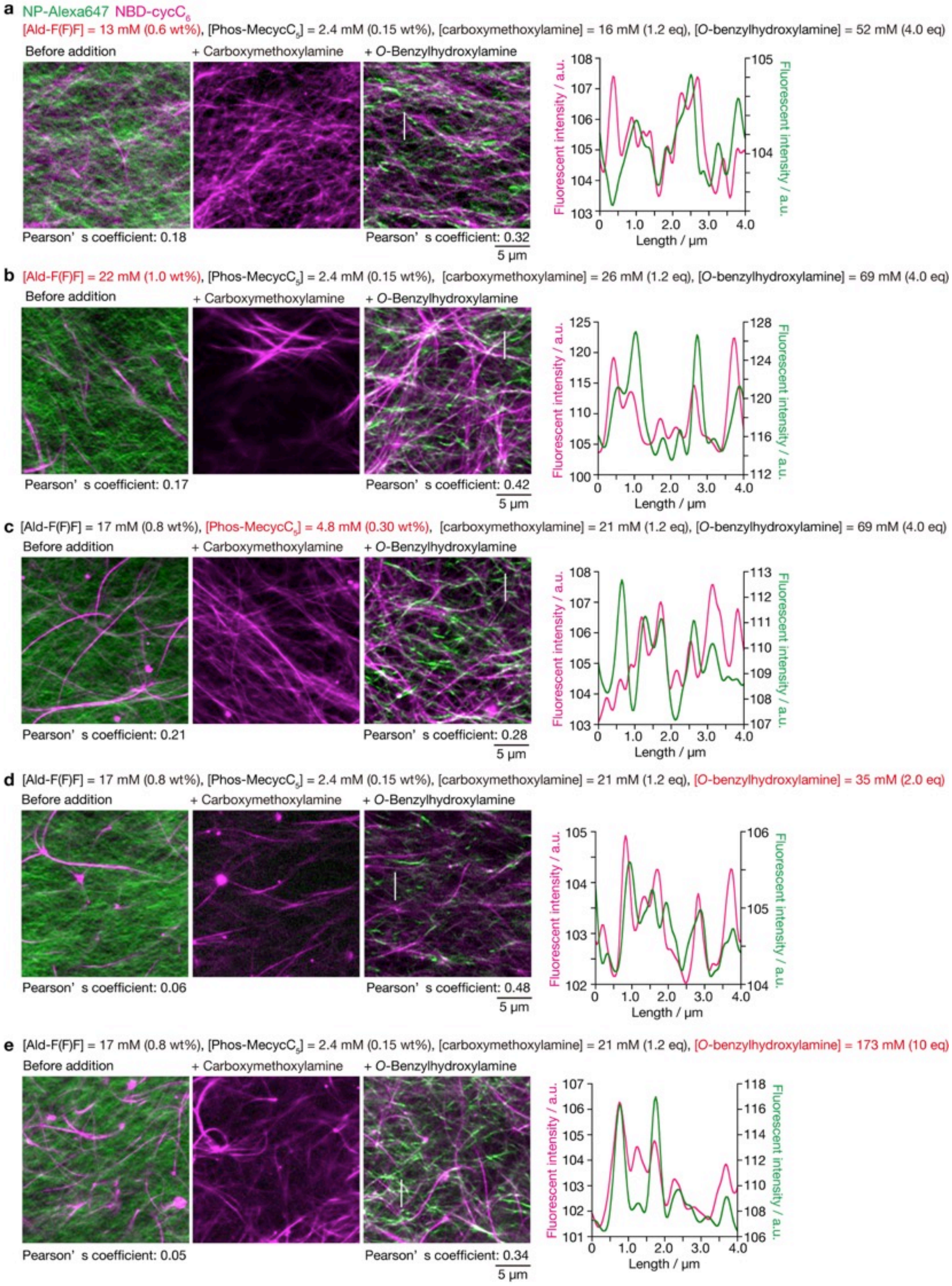

Supplementary Figure 32. High-resolution Airyscan CLSM images of (left) the interpenetrated SDN, (second from the left) the single lipid network, and (third from the left) the parallel SDN under various conditions. (Right) Line plot analysis along white lines shown in the parallel SDN images. Green: **NP-Alexa647**, magenta: **NBD-cycC$_6$**. Condition: 100 mM MES, pH 6.0, 30 ºC.

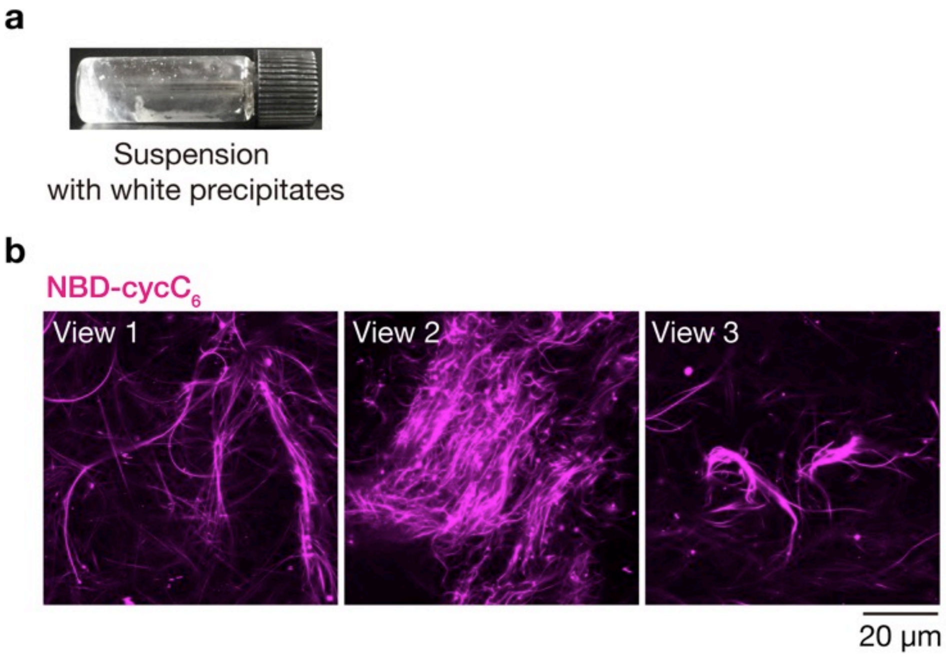

Supplementary Figure 33. (**a**) Macroscopic observation of the mixture of **CaOx-F(F)F** and **Phos-MecycC$_5$**. The sample was prepared by the heat-cool protocol. (**b**) CLSM observation of the mixture of **CaOx-F(F)F**, **Phos-MecycC$_5$**, **NP-Alexa647**, and **NBD-cycC$_6$**. Three different views were shown. There were no fibrous structures stained by **NP-Alexa647**. Condition: [**CaOx-F(F)F**] = 17.3 mM (0.93 wt%), [**Phos-MecycC5**] = 2.4 mM (0.15 wt%) in 100 mM MES, pH 6.0.

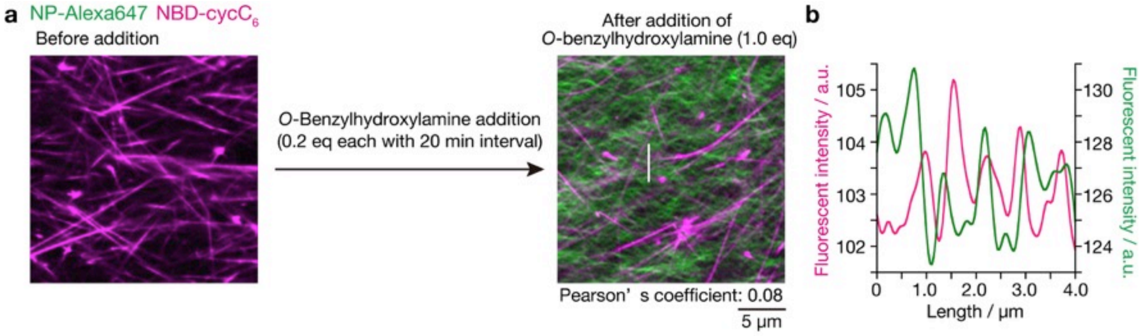

Supplementary Figure 34. (**a**) CLSM imaging of the mixture of **Ald-F(F)F** and **Phos-MecycC$_5$** (left) before and (middle) after treatment of *O*-benzylhydroxylamine. *O*-benzylhydroxylamine was added five times at 20 min intervals (0.2 eq each, 1.0 eq in total). (**b**) Line plot analysis along white lines shown in supplementary Fig. 34a. Green: **NP-Alexa647**, magenta: **NBD-cycC$_6$**. Condition: [**Ald-F(F)F**] = 4.3 mM (0.20 wt%), [**Phos-MecycC$_5$**] = 2.4 mM (0.15 wt%), [*O*-benzylhydroxylamine] = 4.3 mM (1.0 eq), [**NP-Alexa647**] = 4.0 μM, [**NBD-cycC$_6$**] = 4.0 μM, 100 mM MES, pH 6.0, 30 ºC.

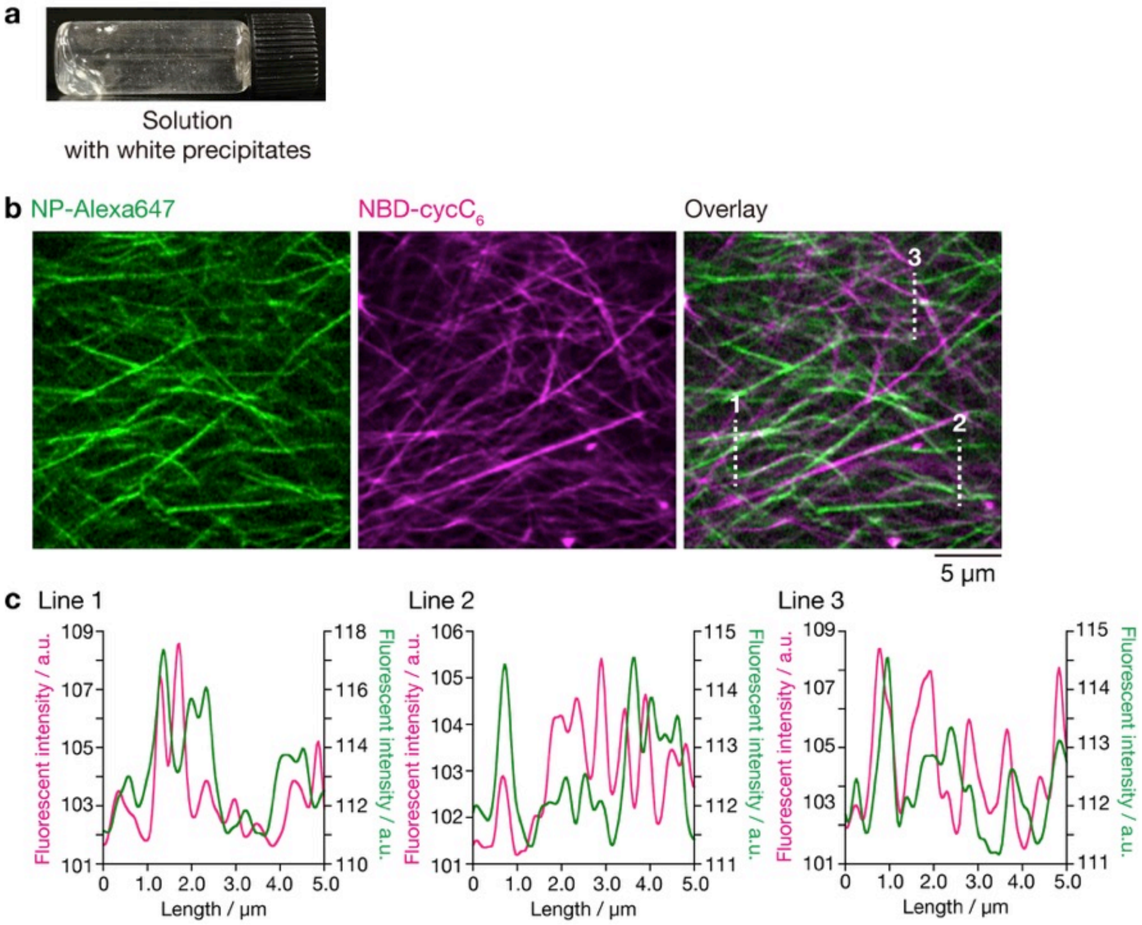

Supplementary Figure 35. (**a**) Macroscopic observation of the mixture of **BnOx-F(F)F** and **Phos-MecycC$_5$**. The sample was prepared by slow cooling of the hot solution of **BnOx-F(F)F** and **Phos-MecycC$_5$**. (**b**) CLSM observation of the mixture of **BnOx-F(F)F**, **Phos-MecycC$_5$**, **NP-Alexa647**, and **NBD-cycC$_6$**. (**c**) Line plot analysis along white lines shown in the overlay image. Condition: [**BnOx-F(F)F**] = 4.3 mM (0.25 wt%), [**Phos-MecycC5**] = 2.4 mM (0.15 wt%), [**NP-Alexa647**] = 4.0 μM, [**NBD-cycC$_6$**] = 4.0 μM in 100 mM MES, pH 6.0.

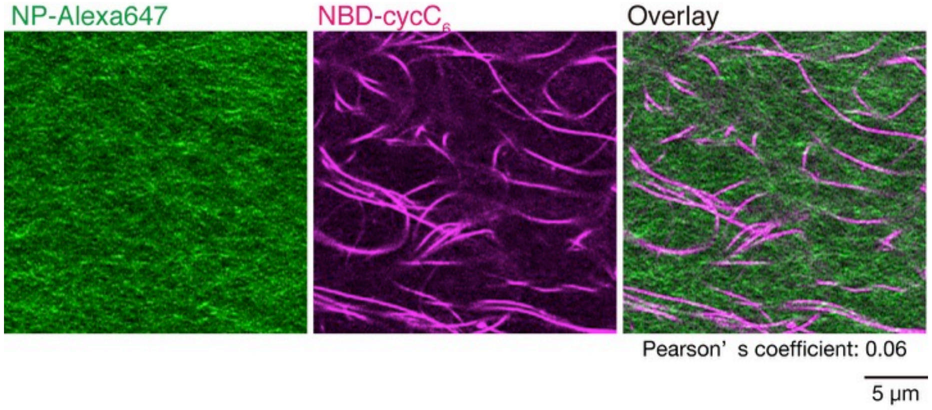

Supplementary Figure 36. CLSM imaging of the hydrogel of **Ald-F(F)F** and **Phos-MecycC$_5$** after addition of *O*-benzylhydroxylamine. Interpenetrated self-sorting network structure retained after addition of *O*-benzylhydroxylamine. Condition: [**Ald-F(F)F**] = 17.3 mM (0.80 wt%), [**Phos-MecycC$_5$**] = 2.4 mM (0.15 wt%), [**NP-Alexa647**] = 4.0 μM, [**NBD-cycC$_6$**] = 4.0 μM, [*O*-benzylhydroxylamine] = 69 mM (4.0 eq) in 100 mM MES, pH 6.0.

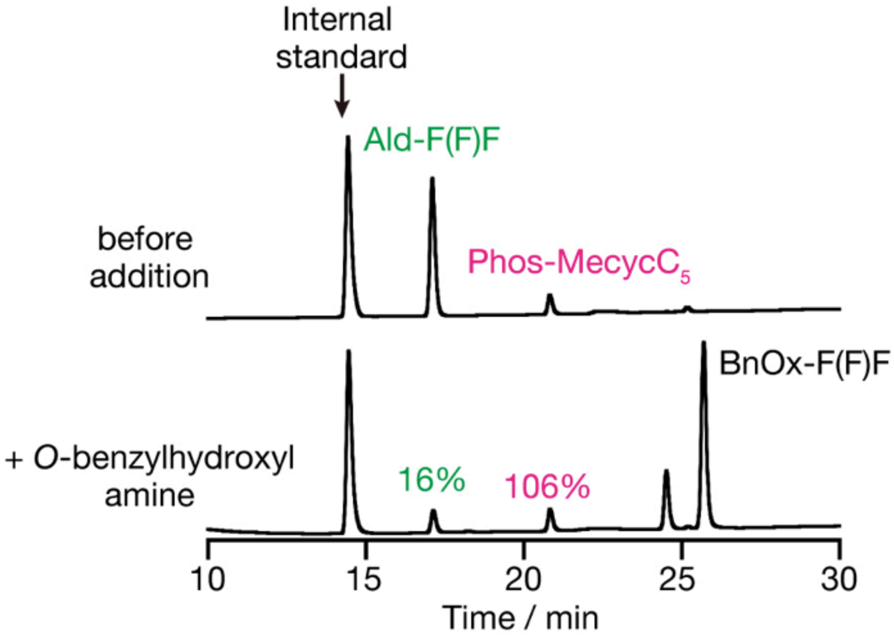

Supplementary Figure 37. HPLC analysis of the interpenetrated hydrogel of **Ald-F(F)F** and **Phos-MecycC$_5$** after addition of *O*-benzylhydroxylamine. Internal standard: fluorescein. The residual ratio of **Ald-F(F)F** was determined by comparing the integral ratio before *O*-benzylhydroxylamine addition as 100%. Condition: [**Ald-F(F)F**] = 17.3 mM (0.80 wt%), [**Phos-MecycC$_5$**] = 2.4 mM (0.15 wt%), [**NP-Alexa647**] = 4.0 µM, [**NBD-cycC$_6$**] = 4.0 µM, [*O*-benzylhydroxylamine] = 69 mM (4.0 eq) in 100 mM MES, pH 6.0.

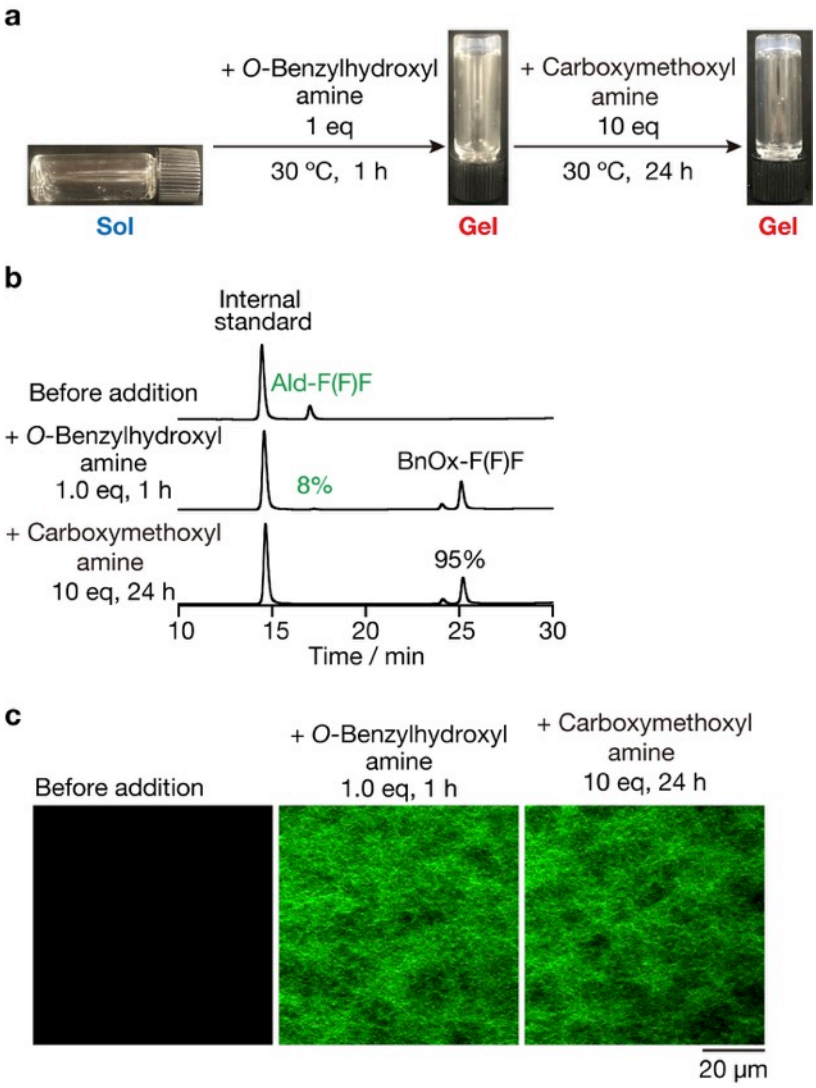

Supplementary Figure 38. (**a**) Macroscopic observation of **Ald-F(F)F** (left) before and after addition of (middle) *O*-benzylhydroxylamine and (right) carboxymethoxylamine. (**b**) HPLC analysis of **Ald-F(F)F** (top) before and after addition of (middle) *O*-benzylhydroxylamine and (bottom) carboxymethoxylamine. Internal standard: fluorescein. The residual ratio of **Ald-F(F)F** and **BnOx-F(F)F** were determined by comparing the integral ratio of **Ald-F(F)F** (the first row) and **BnOx-F(F)F** (the second row) as 100%, respectively. (**c**) CLSM images of **Ald-F(F)F** and **NP-Alexa647** (left) before and after addition of (middle) *O*-benzylhydroxylamine and (right) carboxymethoxylamine. Condition: [**Ald-F(F)F**] = 4.3 mM (0.20 wt%), [*O*-benzylhydroxylamine] = 4.3 mM (1.0 eq), [carboxymethoxylamine] = 43 mM (10.0 eq) in 100 mM MES, pH 6.0.

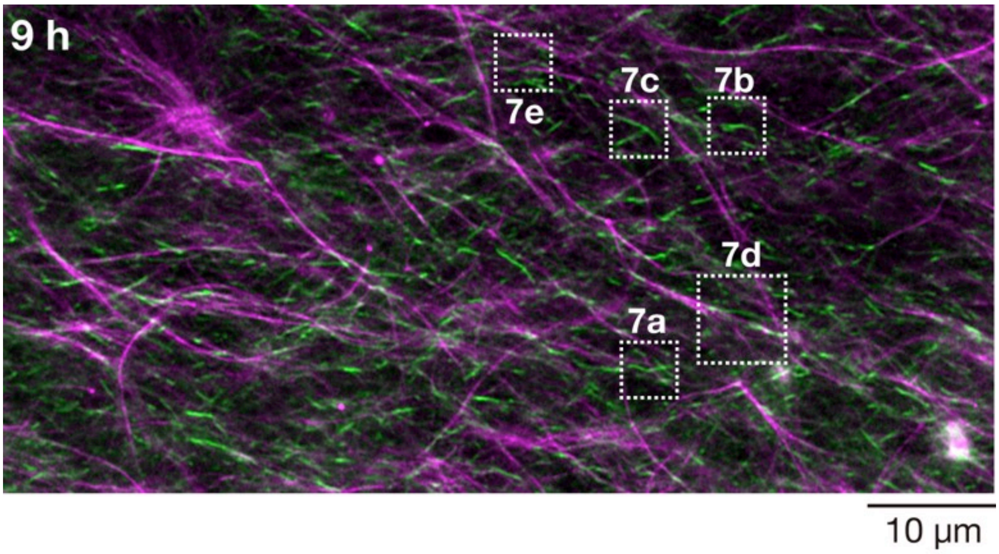

Supplementary Figure 39. The location of unique elongation and collapse behaviors of the peptide-type nanofibers shown in Fig. 7.

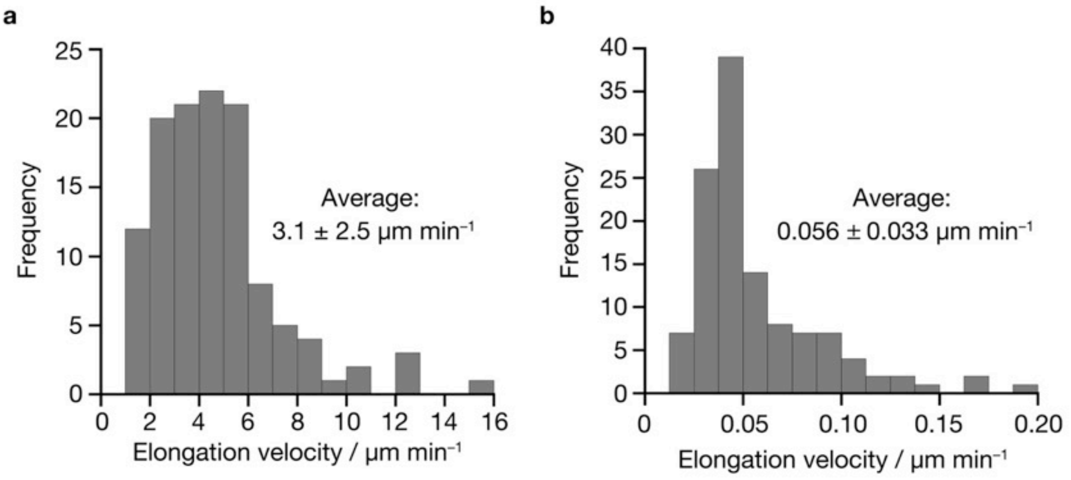

Supplementary Figure 40. Histograms of nanofiber elongation velocity in (**a**) the oxime-formation and (**b**) oxime-exchange protocols. n = 120. The average values represent the mean ± standard deviation.

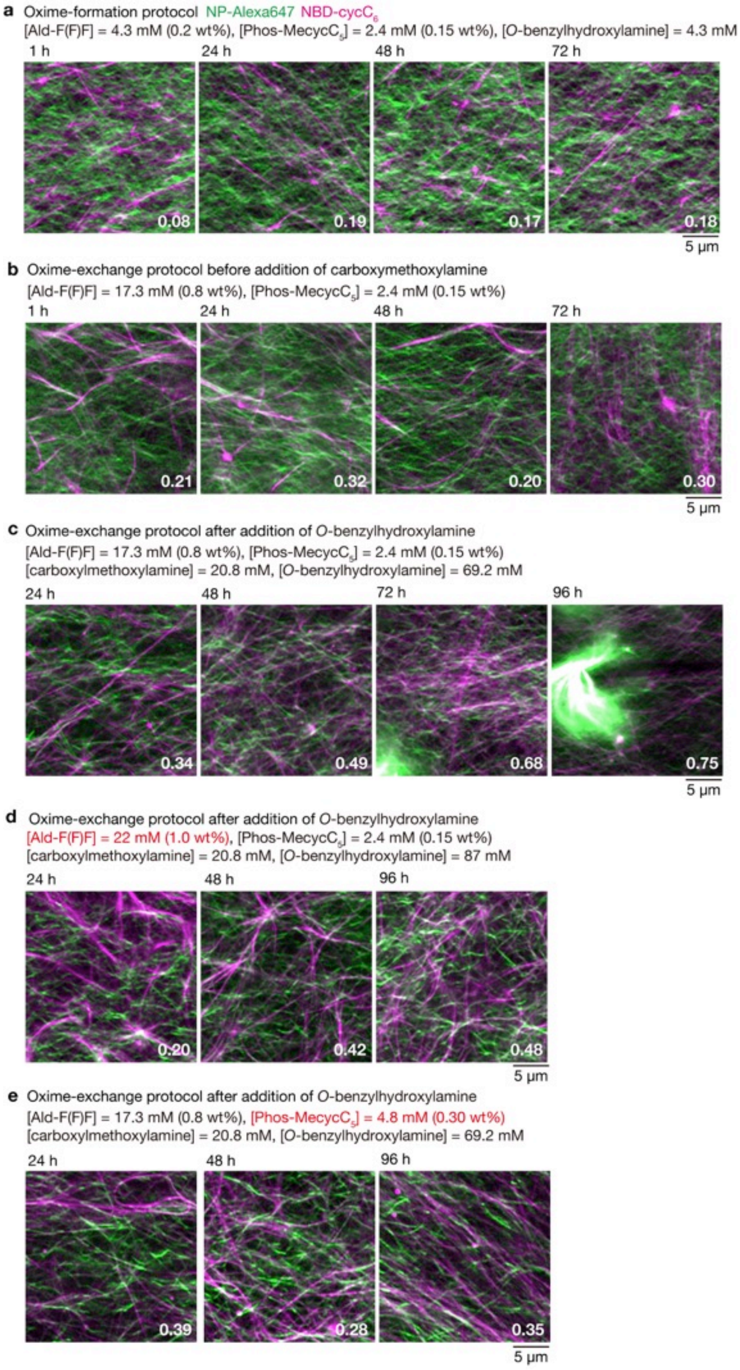

Supplementary Figure 41. Time-dependence of the (**a**,**b**) interpenetrated and (**c**–**e**) parallel SDNs. Pearson's coefficients were shown at the right-bottom of the images. The interpenetrated SDN did not show significant changes after 3 days, while the parallel SDN shown in **c** gradually decomposed to form the coassembled spherical aggregates. In contrast, the parallel SDN became more stable by increment of the concentration of the peptide- or lipid-type hydrogelators as shown in **d** and **e**. Condition: [**NP-Alexa647**] = 4.0 μM, [**NBD-cycC$_6$**] = 4.0 μM in 100 mM MES, pH 6.0.

Supplementary References

1. Hoop, K. A., Kennedy, D. C., Mishki, T., Lopinski, G. P., Pezacki, J. P. Silicon and silicon oxide surface modification using thiamine-catalyzed benzoin condensations. *Can. J. Chem.* **90**, 262–270 (2012).
2. Kubota, R., Liu, S., Shigemitsu, H., Nakamura, K., Tanaka, W., Ikeda, M., Hamachi, I. Imaging-based study on control factors over self-sorting of supramolecular nanofibers formed from peptide- and lipid-type hydrogelators. *Bioconjugate Chem.* **29**, 2058–2067 (2018).
3. Shigemitsu, H., Fujisaku, T., Tanaka, W., Kubota, R., Minami, S., Urayama, K., Hamachi, I. An adaptive supramoleular hydrogel comprising self-sorting double nanofibre networks. *Nat. Nanotechnol.* **13**, 165–172 (2018).
4. Schindelin, J. *et al.* Fiji: an open-source platform for biological-image analysis. *Nat. Meth.* **9**, 676–682 (2012).
