# Supplementary material for: Control of seed formation allows two distinct self-sorting patterns of supramolecular nanofibers
Source: Nat Commun. 2020 Aug 14;11:4100. doi: 10.1038/s41467-020-17984-x (PMC7428048; doi:10.1038/s41467-020-17984-x)
Supplement: Supplementary file 3 — Description of Additional Supplementary Files [file 41467_2020_17984_MOESM3_ESM.pdf]

Description of Additional Supplementary Files

File Name: Supplementary Movie 1
Description: Real-time CLSM imaging of construction of the peptide-type nanofibers (the same as supplementary Fig. 5). Condition: [Ald-F(F)F] = 4.3 mM (0.20 wt%), [O-benzylhydroxylamine] = 4.3 mM (1.0 eq), [NP-Alexa647] = 4.0 µM, 100 mM MES, pH 6.0, 30 ºC. Elapsed time was displayed as mm:ss.

File Name: Supplementary Movie 2
Description: 3D CLSM imaging of the interpenetrated self-sorting network of Ald-F(F)F and Phos-MecycC5 after addition of O-benzylhydroxylamine (the same as supplementary Fig. 10). Condition: [Ald-F(F)F] = 4.3 mM (0.20 wt%), [Phos-MecycC5] = 2.4 mM (0.15 wt%), [NP-Alexa647] = 4.0 µM, [NBD-cycC6] = 4.0 µM, [O-benzylhydroxylamine] = 4.3 mM (1.0 eq) in 100 mM MES, pH 6.0.

File Name: Supplementary Movie 3
Description: Real-time CLSM imaging of construction of the interpenetrated self-sorting network (the same as Fig. 4). Green: NP-Alexa647, magenta: NBD-cycC6. Condition: [Ald-F(F)F] = 4.3 mM (0.20 wt%), [Phos-MecycC5] = 2.4 mM (0.15 wt%), [O-benzylhydroxylamine] = 4.3 mM (1.0 eq), [NP-Alexa647] = 4.0 µM, [NBD-cycC6] = 4.0 µM, 100 mM MES, pH 6.0, 30 ºC. Elapsed time was displayed as mm:ss.

File Name: Supplementary Movie 4
Description: 3D CLSM imaging of the interpenetrated self-sorting network of Ald-F(F)F and Phos-MecycC5 (the same as supplementary Fig. 22). Condition: [Ald-F(F)F] = 17.3 mM (0.80 wt%), [Phos-MecycC5] = 2.4 mM (0.15 wt%), [NP-Alexa647] = 4.0 µM, [NBD-cycC6] = 4.0 µM in 100 mM MES, pH 6.0.

File Name: Supplementary Movie 5
Description: 3D CLSM imaging of the parallel self-sorting network of Ald-F(F)F and Phos-MecycC5 upon treatment of carboxymethoxylamine and O-benzylhydroxylamine (the same as Fig. 5c). Condition: [Ald-F(F)F] = 17.3 mM (0.80 wt%), [Phos-MecycC5] = 2.43 mM (0.15 wt%), [carboxymethoxylamine] = 20.8 mM (1.2 eq), [O-benzylhydroxylamine] = 69.2 mM (4.0 eq), [NP-Alexa647] = 4.0 µM, [NBD-cycC6] = 4.0 µM, 100 mM MES, pH 6.0, 30 ºC.

File Name: Supplementary Movie 6
Description: Real-time CLSM imaging of construction of the parallel self-sorting double network (the same as Fig. 6). Green: NP-Alexa647, magenta: NBD-cycC6. Condition: [Ald-F(F)F] = 17.3 mM (0.80 wt%), [Phos-MecycC5] = 2.43 mM (0.15 wt%), [carboxymethoxylamine] = 20.8 mM (1.2 eq), [O-benzylhydroxylamine] = 69.2 mM (4.0 eq), [NP-Alexa647] = 4.0 µM, [NBD-cycC6] 4 = 4.0 µM, 100 mM MES, pH 6.0, 30 ºC. Elapsed time was displayed as hh:mm.
